# Nanoscale Plasmonic Heating-Induced Spatiotemporal Crystallization of Methylammonium Lead Halide Perovskite

**DOI:** 10.1021/acsnano.5c12057

**Published:** 2025-10-14

**Authors:** Md Shahjahan, Md Ataur Rahman, Sayef Fateure Rahman, Yaqing Zhang, Rihan Wu, Elad Harel

**Affiliations:** Department of Chemistry, 3078 Michigan State University, East Lansing, Michigan 48824, United States

**Keywords:** Laser crystallization, halide perovskites, plasmonic heating, retrograde solubility, spatiotemporal crystallization, high speed imaging

## Abstract

Precise spatiotemporal control over crystallization is essential for advancing optoelectronic materials, yet achieving this in lead halide perovskites remains challenging. Conventional methods rely on preseeded growth, suffer from redissolution, or require complex optical setups. Here, we demonstrate a seed-free, plasmonic heating-driven approach for nanolocalized, on-demand nucleation and growth of methylammonium lead bromide (MAPbBr_3_) perovskites using a continuous-wave (CW) laser. By leveraging localized surface plasmon resonance (LSPR) in gold nanoparticles (AuNPs), we induced supersaturation at the NP surface to initiate crystallization. Using high-speed microscopy, we captured subsequent critical events from nucleation to growth with submillisecond resolution. These insights challenge conventional crystallization models and establish a scalable, mask-free platform for engineering perovskite materials with tailored properties for next-generation optoelectronics.

## Graphical abstract

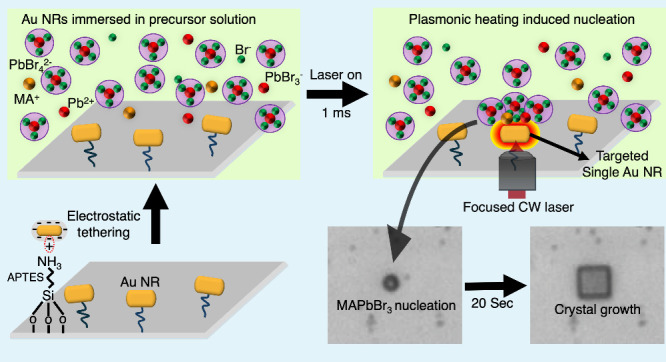

## Introduction

Lead halide perovskites have rapidly advanced as promising materials for next-generation optoelectronics, offering tunable bandgaps, strong light-matter interactions, high photoluminescence quantum yields, and exceptional charge transport properties.^1–8^ Facile synthesis and solution-processing make perovskites attractive for applications in high-efficiency solar cells, light-emitting diodes, lasers, and photodetectors.^9–16^ However, despite advantages in simplicity of synthesis, achieving precise control over perovskite crystallization remains a fundamental challenge. Conventional vapor diffusion or temperature-controlled crystal growth techniques often lead to uncontrolled nucleation at random sites, slow growth rates, and structural heterogeneity, resulting in large variances in crystal orientation, size, and, subsequently, optical properties.^17–22^ These limitations are particularly problematic for integrated photonic applications, where spatially controlled crystallization is critical for optimizing device performance.^22–24^

Achieving spatial and temporal control over perovskite crystallization is essential not only for device integration but also for uncovering fundamental crystallization mechanisms^17,25,26^ that inform the design of materials with tailored properties. To this end, various strategies have been explored, including resonant near-infrared laser heating,^27^ lithographic techniques and laser-assisted crystallization.^28–32^ For example, Chen et al. demonstrated laser-directed optofluidic lithography^29^ to grow MAPbBr_3_ single crystals into arbitrary micropatterns by focusing a 515 nm femtosecond laser onto a seed crystal. Similarly, Yuyama et al. achieved spatiotemporal control of MAPbX_3_ single crystal growth^32^ by leveraging laser trapping in combination with the retrograde solubility of perovskites. However, these approaches rely on seed crystals or suffer from redissolution which restricts precise spatial control over nucleation and crystal growth. Moreover, these methods employ complex optical setups, which limit scalability and flexibility. Therefore, there remains a critical need for a simple yet effective method that enables spatiotemporal control of perovskite crystallization while simultaneously providing insights into nucleation and growth processes.

Here, we demonstrate spatiotemporally controlled growth of MAPbBr_3_ single crystals by leveraging plasmonic heating in gold nanoparticles (AuNPs). Plasmonic heating exploits the unique optical properties of metal nanoparticles^33,34^ to convert light energy into localized heat, enabling precise temperature control in the immediate vicinity of the NP surface.^35–40^ This effect arises from localized surface plasmon resonance (LSPR), where incident light induces a collective oscillation of free electrons, resulting in localized heating.^39,41,42^ Such plasmonic heating has been widely utilized in diverse crystallization methods, where the localized thermal gradients enable precise control over nucleation and growth including polymer, protein, and salts by locally modulating supersaturation conditions.^43–46^ In this study, we utilized a modulated single pulse from a focused 660 nm CW laser, tuned to match the LSPR of optimally sized AuNPs. This targeted excitation generated highly localized heating, driving the nucleation and subsequent growth of MAPbBr_3_ crystals. Further, this method provided a unique opportunity for real-time visualization of the crystallization process with submillisecond resolution using high-speed microscopy. Such insights are key to facilitating the optimization of growth conditions, controlling crystal size and morphology, and enhancing perovskite crystal quality for advanced optoelectronic applications.

## Results and Discussion

A schematic of the laser-induced crystallization of MAPbBr_3_ perovskite single crystals using AuNPs as a plasmonic heating source is shown in Figure 1. A glass substrate (borosilicate), functionalized with AuNPs, is placed under the microscope inside a reaction chamber containing the perovskite precursor solution (Figure 1a).

**Figure 1:**
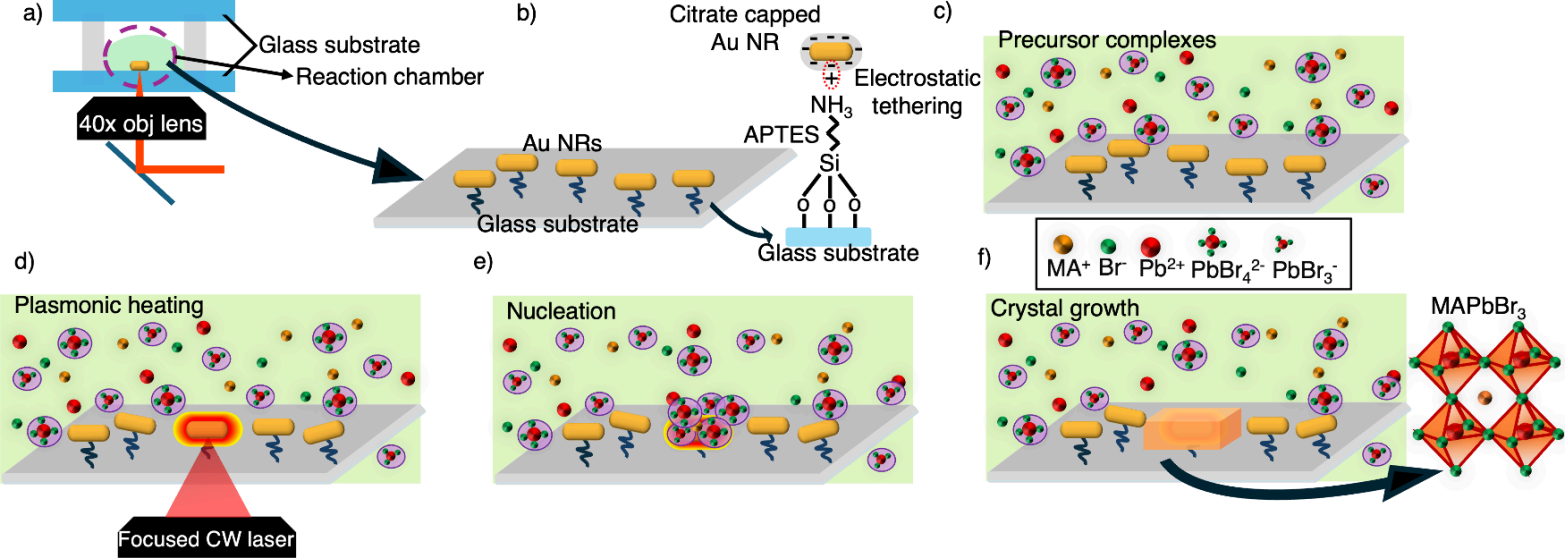
Schematic representation of the laser-induced crystallization process of MAPbBr_3_ using AuNRs. (a) Reaction chamber inside of an inverted microscope (b) Attachment of negatively charged AuNRs on the positively charged APTES-functionalized substrate. (c) View of the reaction chamber containing the MAPbBr_3_ precursor solution. (d) 660 nm CW laser focused onto a single AuNR to induce plasmonic heating. (e) Nucleation of MAPbBr_3_ single crystal at the surface of the AuNR due to localized supersaturation. (f) Natural growth of MAPbBr_3_ crystals from the nucleated sites after the laser has turned off.

The glass substrate is treated with (3-aminopropyl)­triethoxysilane (APTES), which has a silane end that readily binds to the silicon surface, forming a stable anchor. On the other end, it possesses an amine group (−NH_2_), which introduces a positive charge to the surface. This positive charge attracts and holds the negatively charged AuNPs capped with citrate ions that is electrostatically attracted to the positively charged APTES-functionalized glass substrate (Figure 1b).^47–51^ The covalent bonding of APTES to the glass substrate and the subsequent electrostatic attachment of AuNPs prevents the AuNPs from being dislocated due to the thermal stresses induced by the laser irradiation (see Supporting Information (SI) for additional details). For the crystallization of MAPbBr_3_ via plasmonic heating, we prepared the glass substrate by tethering 60 × 25 nm gold nanorods (AuNRs), which exhibit a longitudinal plasmonic frequency maximum near 660 nm that matches with the incident laser wavelength. Optical microscopy and scanning electron microscopy (SEM) images of the prepared AuNRs are shown in Figures S1 and S2. 1.0 M precursor solution of MAPbBr_3_ was then placed on the substrate within the reaction chamber which was sealed with a coverslip to prevent spontaneous crystallization via natural solvent evaporation (Figure 1c). The precursor concentration is slightly lower than what is typically used in spontaneous crystallization methods, where higher concentrations (e.g., 1.3 M) result in uncontrolled nucleation throughout the solution. Detail solution preparation steps and images of spontaneous crystals at higher concentrations are shown in the SI (Figure S3). Subsequently, the targeted AuNRs were illuminated with the 660 nm CW laser with a modulated single (1 ms duration) laser pulse (Figure 1d). When the laser light interacted with the AuNRs, the LSPR effect led to localized heating, resulting in a rapid rise in the temperature, which decays rapidly from the NR surface. This confined temperature increase plays a critical role in modulating the crystallization process, owing to the retrograde solubility behavior of MAPbBr_3_ precursor solution. Unlike typical solute systems where solubility increases with temperature, MAPbBr_3_ exhibits a decrease in solubility with rising temperature up to 100 °C.^19,52^ Consequently, the localized heating causes the precursor solution to become supersaturated near the surface, driving the formation of stable MAPbBr_3_ crystal nuclei by aggregating the dissolved precursor solutes (Figure 1e). Once the nuclei are formed, they act as seeds for further crystal growth (Figure 1f) even after the laser illumination is turned off. The remaining precursor ions then diffuse toward the nucleated site, driven by the concentration gradient. As the ions reach the seeds, they attach to the crystal lattice, causing the crystals to continue to grow. This concentration gradient-driven diffusion of ions allows the crystal to expand well beyond the submicron laser focal spot, reaching several microns in size. Notably, this process is restricted to the localized region influenced by plasmonic heating and enables precise spatiotemporal control over nucleation, while bulk ion diffusion governs the growth of MAPbBr_3_ crystals. The unique combination of LSPR-induced localized heating and retrograde solubility thus provides an effective and scalable strategy for deterministic, seed-free crystallization of MAPbBr_3_ perovskite single crystals.

Figure 2 shows snapshots of the crystallization process by monitoring the interaction of the laser with AuNRs in the precursor solution using brightfield microscopy. Optical microscopy setup equipped with a high-speed camera operating at 2000 frames per second (fps) enabled capture, in real-time, of the morphology evolution with 500 μs temporal resolution. Precise synchronization of the high-speed camera with the laser pulse allowed us to visualize the initial nucleation and the earliest stages of crystal growth that occurred over short time intervals. Figure 2a focuses on the initial nucleation phase, occurring within submilliseconds following laser irradiation. The precise focal spot of the 660 nm CW laser is marked by an orange circle (Figure 2a­(i)), corresponding to the spatial location of a single AuNR tethered to the functionalized glass substrate. Upon laser irradiation, the AuNR undergoes LSPR effect, which rapidly generates confined nanoscale heating. This highly localized heating creates a sharp thermal gradient within the precursor solution which is restricted to the immediate vicinity of the target AuNR. Due to the retrograde solubility of MAPbBr_3_, where solubility decreases with increasing temperature, this localized heat creates a supersaturated microenvironment near the AuNR surface favoring the formation of crystal nuclei precisely at the targeted site. For consistent crystallization of MAPbBr_3_ crystals, the input power of the laser pulse was set at 60 mW, corresponding to a power density of 6.67 × 10^6^ W/cm^2^ at the focal plane. The calculated maximum temperature (*T*_max_) under these conditions is approximately 330 K (see SI and Figure S4 for COMSOL simulation),^29,53,54^ which is sufficient to create supersaturation at the surface of the AuNR to initiate nucleation. By 0.5 ms (Figure 2a-ii), a distinct feature under brightfield microscopy becomes visible at the focal point, indicating the rapid onset of nucleation. As the tracking progresses to 2.0 ms (Figure 2a­(iii–v)), the nucleated site started growing within the first few milliseconds. Figure 2b­(i–-v) extends the observation window spanning from 100 to 500 ms, providing insight into the self-sustaining growth phase of the crystal after nucleation. This growth is driven by the diffusion of dissolved precursor ions toward the nucleation site, initiated by the concentration gradient established during the initial localized heating event. Figure 2c­(i–v) follows the growth of the crystal over several seconds. By 1 s, the crystal adopted a more defined morphology, evolving into a rectangular structure characteristic of typical MAPbBr_3_ crystals. The crystal continues to grow steadily within the 20 s observation time, ultimately forming a well-defined, highly ordered crystal structure. The nucleated crystal continuously grew above the AuNR, attaining a size of ∼3 × 3 μm^2^ after 20 s (Figure 2c­(v)). Images of crystal growth at different time intervals from milliseconds to seconds are presented in Figures S5–S18. Notably, the crystal growth continued beyond the observation window at the same rate as natural growth as long as precursor is available, enabling expansion to much larger sizes over extended times.

**Figure 2:**
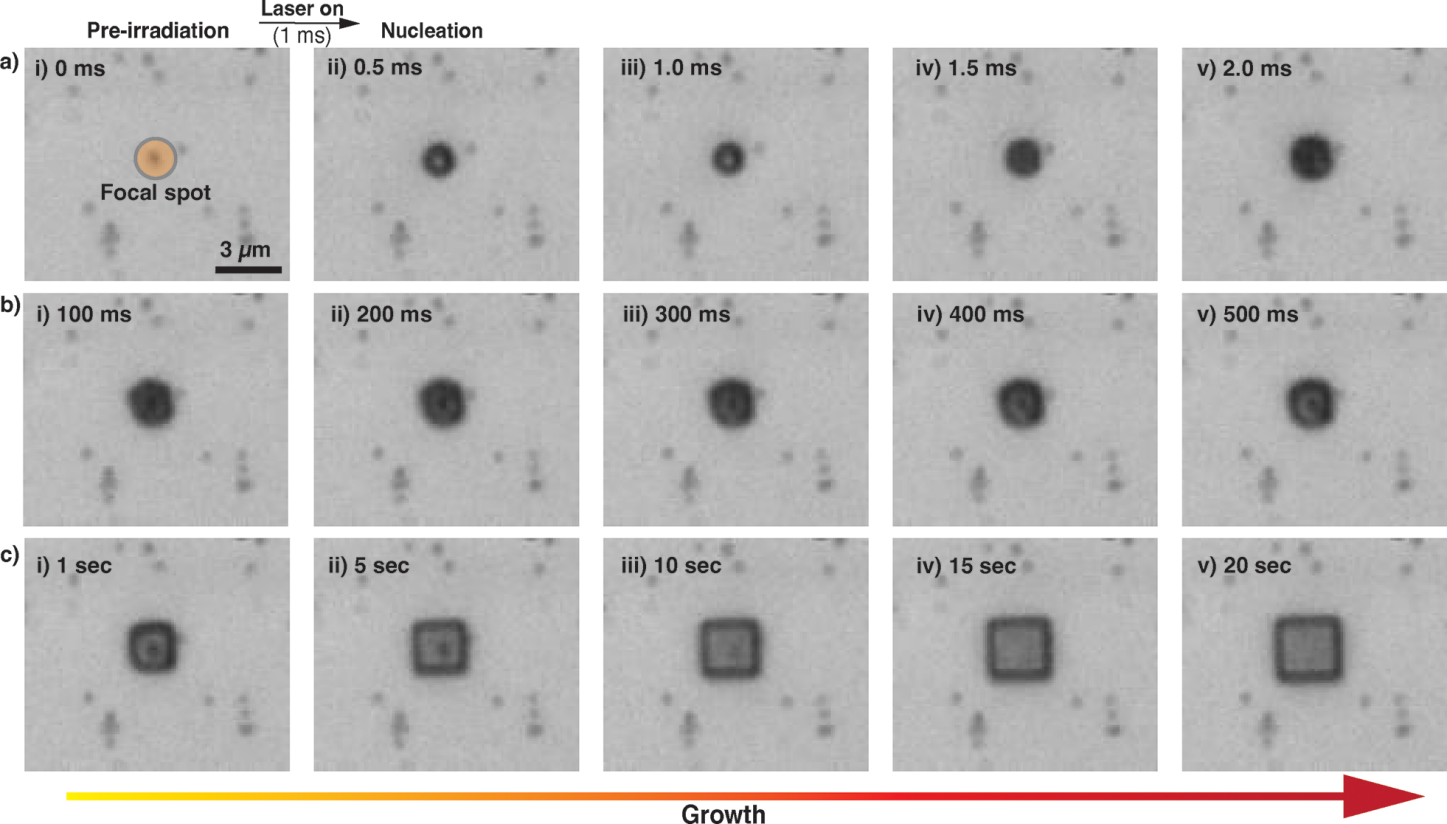
Optical microscopy images capturing the plasmonic heating-induced crystallization progress of MAPbBr_3_ crystals. (a) High-speed imaging of the initial nucleation event occurring at the AuNR site upon laser irradiation. (i) The orange circle marks the focal spot of the 660 nm CW laser. (ii–v) Initial nucleation phase within 0.5 ms of laser activation, with continued growth observed up to 2.0 ms. (b) (i–v) Crystal growth progression from 100 to 500 ms, driven by diffusion of precursor ions toward the nucleated site. (c) (i–v) Crystal growth tracking from 1s to 20s, showing the evolution of a well-defined MAPbBr_3_ single crystal above the AuNR.

**Figure 3:**
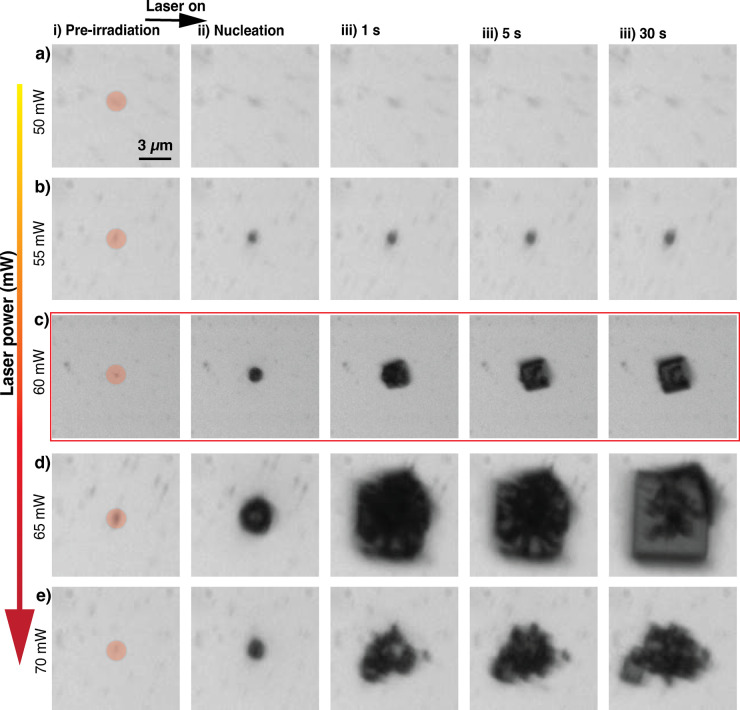
Optical images of MAPbBr_3_ crystallization at varying laser power. (a) No crystallization at 50 mW. (b) Limited nucleation at 55 mW with no significant growth. (c) Optimized nucleation and growth at 60 mW. (d) Faster nucleation and growth at 65 mW, resulting in disordered crystals. (e) Uncontrolled nucleation at 70 mW leading to multiple small crystals, illustrating the critical influence of laser power on crystallization. (a–e) (i) Images of the target AuNR (orange circle) in the precursor solution. (ii) Nucleation of MAPbBr_3_ crystals at the focal spot after laser irradiation. (iii–v) Growth of MAPbBr_3_ crystals over time, showing the development of crystal size and morphology at 1, 5, and 30 s.

Besides thermal effects, plasmonic nanoparticles can generate nonthermal effects such as hot electron injection or local field enhancement under laser illumination. Such nonthermal pathways are unlikely under our conditions. We measured the absorption spectrum of MAPbBr_3_ and found its absorption edge at ∼526 nm (Eg ≈ 2.36 eV). The conduction band minimum (CBM) of MAPbBr_3_ lies at approximately −3.7 eV relative to vacuum, while the Au Fermi level is approximately −5.1 eV. Therefore, for an electron from the Au nanorod’s Fermi level to be transferred into the perovskite’s conduction band, it needs to gain at least 1.4 eV of energy. Although 660 nm laser excitation (1.88 eV), excite collective plasmon modes that generate a nonequilibrium hot-electron distribution, only a very small fraction of carriers attains sufficient energy to cross this energy barrier. Moreover, ultrafast electron–electron and electron–phonon scattering in Au occurs on the subpicosecond time scale, much faster than interfacial transfer, causing most hot carriers to thermalize before injection. As a result, hot-electron transfer is both energetically marginal and kinetically suppressed under our experimental conditions. To verify whether nonthermal plasmonic effects could contribute to MAPbBr_3_ nucleation, controlled experiments were conducted using CsPbBr_3_ precursors in DMF/GBL, a solvent system in which CsPbBr_3_ exhibits normal solubility (i.e., solubility increases with temperature). In contrast to MAPbBr_3_, which shows retrograde solubility and undergoes nucleation under local plasmonic heating, CsPbBr_3_ did not nucleate under identical single-nanorod illumination conditions (Figure S19). These results support our conclusion that MAPbBr_3_ crystallization is thermally driven via retrograde solubility, and not by nonthermal plasmonic processes. This sequence confirms that plasmonic heating enables seed-free crystallization with precise spatial and temporal control, while high-speed imaging reveals the nucleation and subsequent crystal growth process.

To better understand the influence of plasmonic heating on crystal growth, we varied the laser power from 50 to 70 mW in increments of 5 mW. Figure 3 shows how subtle changes in laser power directly control the crystallization process of the resulting MAPbBr_3_ crystals. At the lowest power (50 mW), no crystallization or growth was observed after laser irradiation (Figure 3a). This occurred because the energy input is insufficient to raise the local temperature above the nucleation threshold, preventing supersaturation and nucleation. Upon increasing laser power (55–65 mW), enhanced plasmonic heating at the AuNRs generated a localized temperature gradient, inducing controlled supersaturation that facilitated nucleation and directed the growth of MAPbBr_3_ crystals.

At 55 mW, localized heating induced supersaturation near the AuNRs, leading to the formation of crystal nuclei. However, the limited heat generated at this power resulted in a less steep concentration gradient, reducing the thermodynamic driving force for the diffusion of precursor species toward the nucleated crystal. As a result, while nucleation occurred after laser irradiation (Figure 3b­(ii)), further growth of the nucleated area was not observed up to 30 s due to an insufficient precursor concentration (Figure 3b­(ii–v)). We found that in a narrow range around 60 mW, there is an optimal thermal environment at the focal spot, whereby MAPbBr_3_ single crystals nucleate and continue to grow steadily (Figure 3c). Images of crystal growth at 1s intervals up to 30 s are presented in Figure S20. Increasing the laser power to 65 mW resulted in faster nucleation and more rapid crystal growth due to the higher degree of localized supersaturation (Figure 3d­(ii–v)). However, this rapid nucleation limited the time for precursor molecules to arrange into an ordered crystal lattice, leading to defects or dislocations. At even higher power (70 mW), excessive heat generation lead to uncontrolled nucleation (Figure 3e­(ii)), resulting in the formation of multiple small crystals (Figure 3e­(iii–v)), hindering the development of a well-defined crystal structure. The presence of multiple nucleation centers at 70 mW power leads to competition for available precursor ions, thereby resulting in smaller initial nuclei. In contrast, at 65 mW, the localized heating is sufficient to drive nucleation at a single dominant site, but the excessive thermal energy leads to the formation of a larger yet defect-prone nucleation site rather than multiple, smaller nuclei. Another set of laser-induced MAPbBr_3_ crystallization at varying laser power (50–70 mW) is presented in Figure S21. This observation highlights the critical role of laser power in controlling not only the nucleation process but also the subsequent growth of crystals in plasmon-induced crystallization. We selected 60 × 25 nm AuNRs for our experiments because their peak wavelength at ∼660 nm matches directly with the 660 nm excitation wavelength of our laser, which ensure maximal absorption and efficient local heating. However, the approach is not limited to a specific AuNP size. For example, we also prepared glass substrates with 100 nm Au urchins (Figure S22) following the similar APTES-functionalization. Absorption spectra of both the NPs are shown in Figure S23. The 100 nm Au urchins exhibit a broad plasmon resonance centered near 660 nm, and we observed MAPbBr_3_ crystallization (Figure S24) under 660 nm excitation. This demonstrates that the strategy can be readily extended to other Au nanostructures by selecting particle geometries whose plasmon resonance overlaps the excitation wavelength.

To comprehensively investigate the optical properties of laser-grown MAPbBr_3_ crystals compared to the naturally grown counterparts, we employed two complementary photoluminescence (PL) and Raman spectroscopic characterization approaches presented in Figure 4, shedding light on their structural and vibrational properties. We note that it is not possible to perform XRD on these small crystals inside of the reaction chamber, necessitating an all-optical, in situ characterization approach. For wide-field PL imaging, a 455 nm LED was used to uniformly illuminate the entire MAPbBr_3_ crystals, enabling rapid visualization of overall emission characteristics. Under the 455 nm LED excitation, both laser-grown and naturally grown crystals show intense green emission with a peak maximum at 535 and 533 nm respectively due to their characteristic band-to-band electronic transitions (Figure 4a,b­(i)).^55,56^ The overall PL spectra of the crystals are shown in Figures S19 and S20­(a). However, wide-field PL imaging integrates signals across the entire illuminated area, making it less sensitive to localized variations or subtle inhomogeneities within the crystals. To overcome this limitation and achieve high spatial resolution, we performed point-by-point PL intensity mapping using a confocal microscope with a 462 nm laser source.

**Figure 4:**
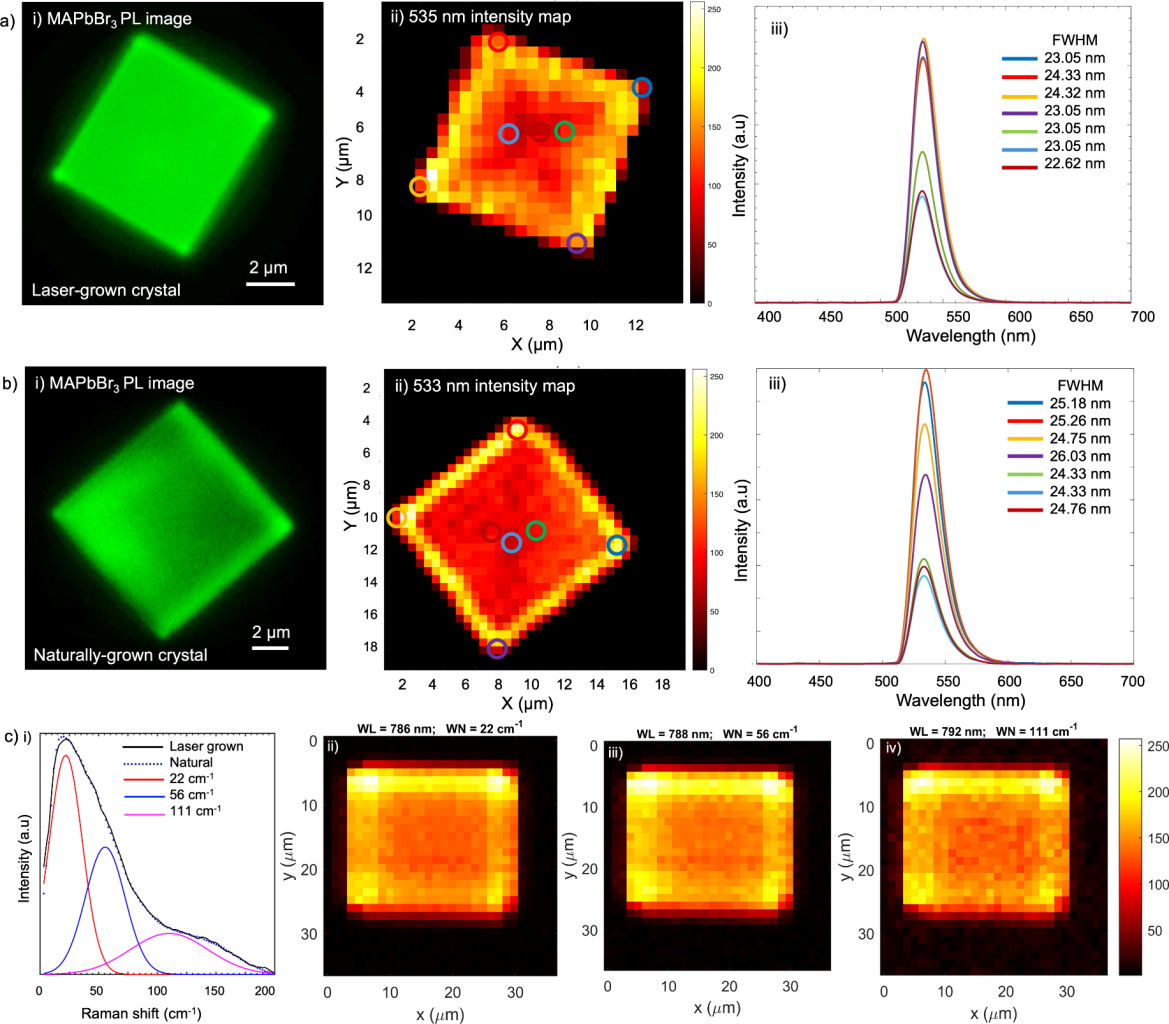
Photoluminescence (PL) and Raman spectroscopic characterization of laser-grown and naturally grown MAPbBr_3_ microcrystals. (a, b) (i) Wide-field PL image of the laser-grown and naturally grown MAPbBr_3_ crystals under 455 nm LED excitation, exhibiting characteristic green emission. (a, b) (ii) Corresponding PL intensity mapping at 535 and 533 nm obtained via 462 nm confocal laser scanning with 1 μm lateral resolution, revealing subtle changes across the crystals with localized edge enhancement. (a, b) (iii) Spatially resolved PL intensity fluctuations measured at marked points across both the crystals, indicating intensity variance between bulk and edges. (c) (i) Low-frequency Raman spectra of both laser-grown and naturally grown MAPbBr_3_ crystals, showing distinct vibrational modes at 22, 56, and 111 cm^–1^. (c) (ii–iv) Spatially resolved Raman intensity map of the laser-grown MAPbBr_3_ crystal at 22, 56, and 111 cm^–1^, obtained via spatial point scanning with 0.8 μm resolution.

The crystals were scanned with a lateral resolution of 1 μm. Recording the PL spectra at each point generated spatially resolved PL intensity maps. This confocal approach offers several distinct advantages. First, it allows for selective excitation and collection of PL spectra from a limited volume, minimizing background fluorescence, and scattered light from regions outside the focal plane. Second, by scanning the excitation spot across the crystal in a controlled manner, we obtained high-resolution PL maps that reveal subtle variations in emission intensity and uniformity that are often compromised in wide-field PL images. Figure 4a, b­(ii) shows PL intensity mapping at 535 and 533 nm for the laser-grown and naturally grown crystals, respectively. In both MAPbBr_3_ crystals, the PL mapping revealed enhanced PL intensity at the crystal edges compared to the bulk. The enhanced PL at the edges suggests that these regions possess fewer nonradiative recombination centers or are better passivated compared to the bulk, leading to more efficient radiative recombination. Similar edge-enhanced emission has been reported in perovskite crystals and nanostructures, attributed to lower defect density, surface passivation, and strain relaxation at the boundaries.^57–59^ Moreover, the observed PL intensity fluctuations within the bulk indicate underlying structural or compositional heterogeneity, such as halide inhomogeneity, strain variations, or nanoscale domain formation, all of which can introduce spatial variations in defect density and recombination dynamics.^60,61^ These variations are primarily attributed to the nonuniform distribution of intrinsic and extrinsic defects e.g., point defects, deep-level traps, which act as nonradiative recombination centers and reduce PL intensity.^62^ Consistent with the full width half maxima (FWHM), the PL mapping shown in Figures S25 and S26­(d–f) at three different wavelengths 540, 550, 560 nm exhibit almost similar mapping all over the crystals. Also, the intensity maps at 500 and 600 nm shown in Figures S25 and S26­(b,c), which lie outside the PL emission bandwidth, show minimal or no signal across the crystals, indicating the absence of spectrally resolved emission at these wavelengths. Figure 4a, b­(iii) illustrates the intensity fluctuations over marked points within the laser-grown and natural crystals. These plots show PL intensity collected from edge and bulk regions, represented by colored circles in the corresponding PL image. From the spectra it is obvious that the PL intensity varies from edges to bulk along with the fluctuations in FWHM by 1–2 nm. Notably, the laser-grown crystal shows slightly narrower PL spectra compared to the natural grown crystal although further studies are needed to quantify differences in crystal quality. To further investigate structural characteristics of the lead-halide octahedral framework, low-frequency Raman spectra of laser-grown and naturally grown MAPbBr_3_ crystals are compared in Figure 4c. The spectrum of both crystals revealed enhanced low-frequency vibrational modes, particularly at 22 cm^–1^, 56 cm^–1^, and 111 cm^–1^ (Figure 4c­(i)). These observed vibrational modes align well with literature reports on cubic MAPbBr_3_ perovskites, where Raman-active phonon bands in the range of 18–150 cm^–1^ are typically associated with Pb–X lattice dynamics and interactions with organic cations.^63–65^ Spatially resolved Raman intensity map of the laser-grown MAPbBr_3_ at wavenumber 22, 56, and 111 cm^–1^ is plotted in Figure 4c­(ii–iv), acquired with a 0.8 μM lateral resolution. Intensity map at 22, 56, and 111 cm^–1^ Raman bands of the laser-grown crystal, deconvoluted spectra and intensity maps of the naturally grown crystal are shown in Figure S27. The comparable PL emission images, spectra, and Raman vibrational modes observed for both laser-induced and naturally grown MAPbBr_3_ crystals confirm that the crystallization process driven by localized plasmonic heating yields high-quality crystals with spectroscopic characteristics comparable to the naturally grown counterparts.

## Conclusion

In this study, we developed a simple yet powerful approach for spatiotemporally controlled crystallization of MAPbBr_3_ perovskite crystals using plasmonic heating from gold nanoparticles (AuNPs). By harnessing localized surface plasmon resonance (LSPR), we precisely control heat generation at the AuNPs, inducing localized supersaturation and nucleation of MAPbBr_3_ crystals. High-speed imaging unveiled the crystallization pathway from nucleation to ordered crystalline phase. Our findings establish plasmonic heating as a precise tool for controlling crystallization dynamics, offering new strategies for optimizing perovskite synthesis. Beyond perovskites, this insight into crystallization progression provides a broader framework for understanding crystallization in other material systems. This work paves the way for scalable, high-quality perovskite fabrication, enhancing their applicability in industrial and commercial optoelectronic technologies.

## Materials and Methods

### Materials

The following commercially available chemicals and materials were used in the study; methylammonium bromide, MABr (Sigma-Aldrich, >98.0%), lead­(II) bromide, PbBr_2_ (Sigma-Aldrich, ≥98.0%), N,N-dimethylformamide, (DMF) (Wako), gamma-butyrolactone, (GBL) (TCI), 1 M sodium hydroxide (NaOH) solution, Milli-Q water, ethanol, piranha solution (H_2_SO_4_:H_2_O_2_, 5:1), (3-aminopropyl) triethoxysilane (APTES), 60 nm × 25 nm AuNRs (nanoComposix), cover glass (25 mm × 25 mm, thickness 0.13–0.17 mm), imaging spacer, (1 well, 9 mm × 0.12 mm) (Grace biolabs secureseal). The precursors and solvents were used as received without any further purification.

### SEM Characterization

Morphologies of the AuNPs were measured using an ultrahigh resolution JEOL 7500F SEM with a cold field emission emitter equipped with Oxford EDS systems.

### UV–Vis Measurements

The UV/vis absorbance spectra of the nanoparticles were recorded in direct transmission mode using a Cary 60 UV–vis spectrophotometer (Agilent technologies)

### Optical Setup for Laser-Induced Crystallization, Bright-Field Imaging, and PL Analysis

The optical setup was designed for laser-induced crystallization, bright-field imaging, and photoluminescence (PL) analysis by integrating multiple optical components. For the laser-induced crystallization a CW laser source of 660 nm wavelength (Cobolt-06-MLD, HUBNER photonics) is first directed into a spatial filter system, which consists of a telescope arrangement made up of two convex lenses with focal lengths of f = 50 mm and f = 250 mm. The laser beam initially passes through the 50 mm lens, which focuses it onto a 100 μm pinhole placed at its focal plane. This spatial filter removes unwanted higher-order beam distortions and ensures a clean Gaussian beam profile. After passing through the pinhole, the beam is collimated by the second lens (f = 250 mm), producing a uniform beam with well-defined intensity distribution. Once collimated, the laser beam is directed onto a 45° angled mirror, which reflects it toward the microscope objective lens (RMS40X-PF-40X Olympus Plan Fluorite Objective, 0.75 NA, 0.51 mm WD). The objective lens focuses the laser tightly onto the sample stage, ensuring highly localized heating for laser-induced crystallization. The reflected portion of the laser beam is collected by the same objective lens and then guided through a beam splitter, which directs it toward a focusing lens (f = 200 mm) before reaching the camera.

For bright-field imaging, a 455 nm fiber LED (M455F3-455 nm, 17 mW (Min) Fiber-Coupled LED, 1000 mA, SMA) light source is introduced from the top of the setup using a Köhler illumination system. The LED light is first diffused and then passed through a microscope objective lens (RMS40X-PF - 40X Olympus Plan Fluorite Objective, 0.75 NA, 0.51 mm WD), which directs it uniformly onto the sample. The transmitted light from the sample is then collected by the bottom objective lens, following the same optical path as the laser-reflected light. The transmitted image is processed through a beam splitter and refocused by a f = 200 mm lens onto the camera. Using a 500 nm long pass filter, the wide-field PL imaging was collected by the top camera. The filtered PL emission is directed toward a color CMOS camera (AmScope FMA050) for PL imaging.

For point-by-point photoluminescence (PL) analysis, another CW laser source (Lasertack GmbH, LDM-462-1400-C, λ = 462 nm) is introduced. This laser, like the laser used for crystallization, is first passed through a spatial filter system to ensure a clean, well-defined Gaussian beam profile. The spatial filter consists of a telescope system with two lenses and a 50 μm pinhole placed at the focal plane of the first lens. After filtering, the collimated 462 nm beam is directed through a series of mirrors, which carefully align and direct it toward the objective lens. Before reaching the objective, the beam encounters a 490 nm dichroic mirror, which reflects the 462 nm excitation laser toward the sample while allowing longer-wavelength 660 nm laser beam to pass through. Once the sample is illuminated, the PL emission is collected by the same objective lens. The emission is then filtered by a 500 nm long-pass filter, which removes laser light and isolates the PL signal.

PL spectra of the perovskite crystals are recorded using a spectrometer (Ocean Optics, Flame). After excitation with the 462 nm wide-field laser, the resulting PL emission from the perovskite microcrystals is collected by the bottom objective lens that was used for 660 nm laser-irradiation. Once collected, the PL signal is transmitted through a 500 nm long-pass filter placed in the detection path to block the excitation laser wavelength while allowing only the emitted PL signal to pass through. This filtered PL signal is then focused onto the spectrometer for PL spectra analysis.

### Raman Spectra Measurements

The Raman spectra of the prepared microcrystals were obtained using our home-built THz Raman Microscope with an excitation laser of 785 nm continuous wave laser (Coherent Ltd.). The beam was focused onto a diffraction limited spot of the sample using a 50X/0.42 NA objective (Mitutoyo). For spectral analysis, after illuminating the microcrystals, the reflected light from the samples were efficiently collected which passes through a noise block beam splitter (785 nm cutoff frequency) and is directed to a high-resolution spectrometer (FERGIE, Princeton Instruments) through a SureBlock XLF (Ondax, Double notch filter system that blocks 785 nm light).

Raman imaging was performed by point-by point spatial scanning using the same 785 nm excitation wavelength. We conducted 2800–3400 spatial point scanning depending on the crystal size with a 0.8 μM resolution. The spatial arrangement of the microcrystals was guaranteed by placing the sample on a high-resolution motorized XY stage provided by Physik Instrumente.

## Supplementary Material



## Notes

Preparation of the Au functionalized cover glasses, synthesis of MAPbBr_3_ precursor solution for laser-induced crystallization, synthesis of MAPbBr_3_ microcrystals via natural solvent evaporation, COMSOL simulations, time-lapse of bright-field images, crystallization at varying laser power, spectral and spatial characterization of natural and laser-grown MAPbBr_3_ crystals, Raman spectra and spatial Raman mapping of natural and laser-grown MAPbBr_3_ crystals (PDF)
