# Supplementary material for: Nanoscale Plasmonic Heating-Induced Spatiotemporal Crystallization of Methylammonium Lead Halide Perovskite
Source: ACS Nano. 2025 Oct 14;19(43):37910–8. doi: 10.1021/acsnano.5c12057 (PMC12593378; doi:10.1021/acsnano.5c12057)
Supplement: Supplementary file 1 [file nn5c12057_si_001.pdf]

# Supporting information

## Nanoscale Plasmonic Heating Induced Spatiotemporal Crystallization of Methylammonium Lead halide Perovskite

Md Shahjahan, Md Ataur Rahman, Sayef Fateure Rahman, Yaqing Zhang, Rihan Wu, and Elad Harel*

Department of Chemistry, Michigan State University, East Lansing, MI 48824

Email: elharel@msu.edu

**Preparation of the Au functionalized cover glasses**

Silicon substrates with 22 × 60 mm size were ultrasonically cleaned in 1 M sodium hydroxide (NaOH) solution, Milli-Q water, and ethanol for 30 min, respectively. The clean glass slides were immersed in piranha solution ($H_2SO_4$:$H_2O_2$, 5:1) for 10 min to decorate the glass substrate with hydroxyl groups (OH-). The clean glass slides were then incubated in 5% (V/V) (3-aminopropyl) triethoxysilane (APTES) in ethanol for 3h. After 3h the cover glasses were washed with ethanol and dried under $N_2$ stream. The slides were then subjected to thermal annealing in a vacuum oven at 110 °C for 2 h to obtain APTES-silanized slides with amine groups. The amino groups on the APTES molecules are used to immobilize AuNPs onto the substrate due to the specific affinity of the amino group to the colloidal gold nanoparticles.

To control the density of AuNRs on the APTES functionalized, the as-received AuNR solution (60 nm × 25 nm AuNRs, nanoComposix) was diluted in Milli-Q water to different concentrations (1x and 10x). The diluted solutions were then sonicated for 30 minutes to ensure uniform dispersion. Subsequently, the diluted suspensions were deposited onto the APTES-functionalized cover glass substrates and allowed to adsorb for 6 h. After adsorption, the samples were washed to remove loosely bound particles and dried in an oven at 50 °C for 2 h.

Representative optical microscopy and SEM images with 1x and 10x dilution are shown in Figure S1 and S2. At higher concentrations (1x dilution), particles appear clustered with sub-micron separations, whereas at lower concentrations (10x dilution) they appear well isolated. The distance between the particles in the representative SEM images (Figure S2 (c,d)) were calculated using ImageJ analyzer. The average center-to-center distances between the particles are well beyond the diameter of the laser focal spot which is ~0.45 μm FWHM at 660 nm with 0.75 NA. Under these optimized 10x dilution, single-particle excitation is ensured for controlled plasmonic heating-induced crystallization.

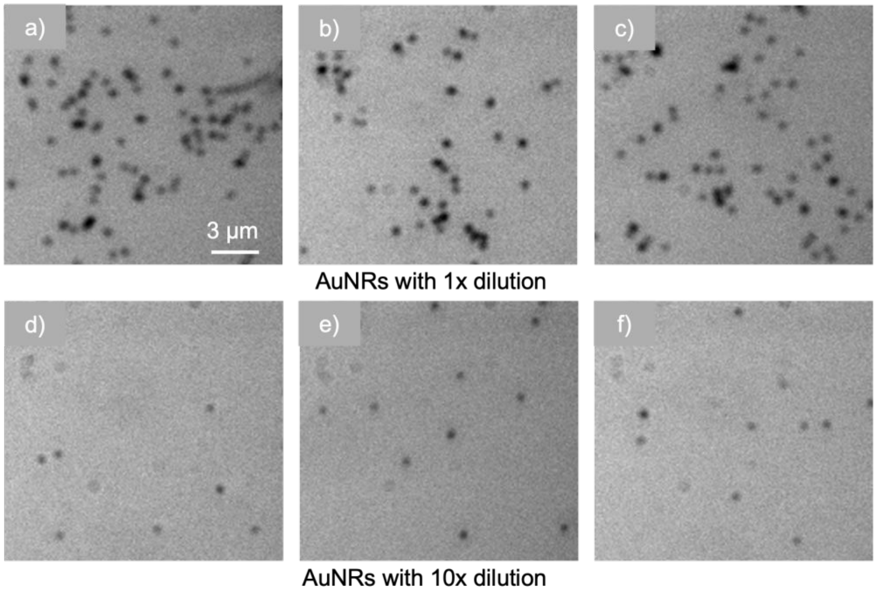


**Figure S1:** Optical microscopy images showing AuNRs coverage at different dilutions of the as-received colloid. (a-c) At 1x dilution, AuNRs appear in clusters or aggregates, while 10x dilution (d-f) results in well-dispersed particles.

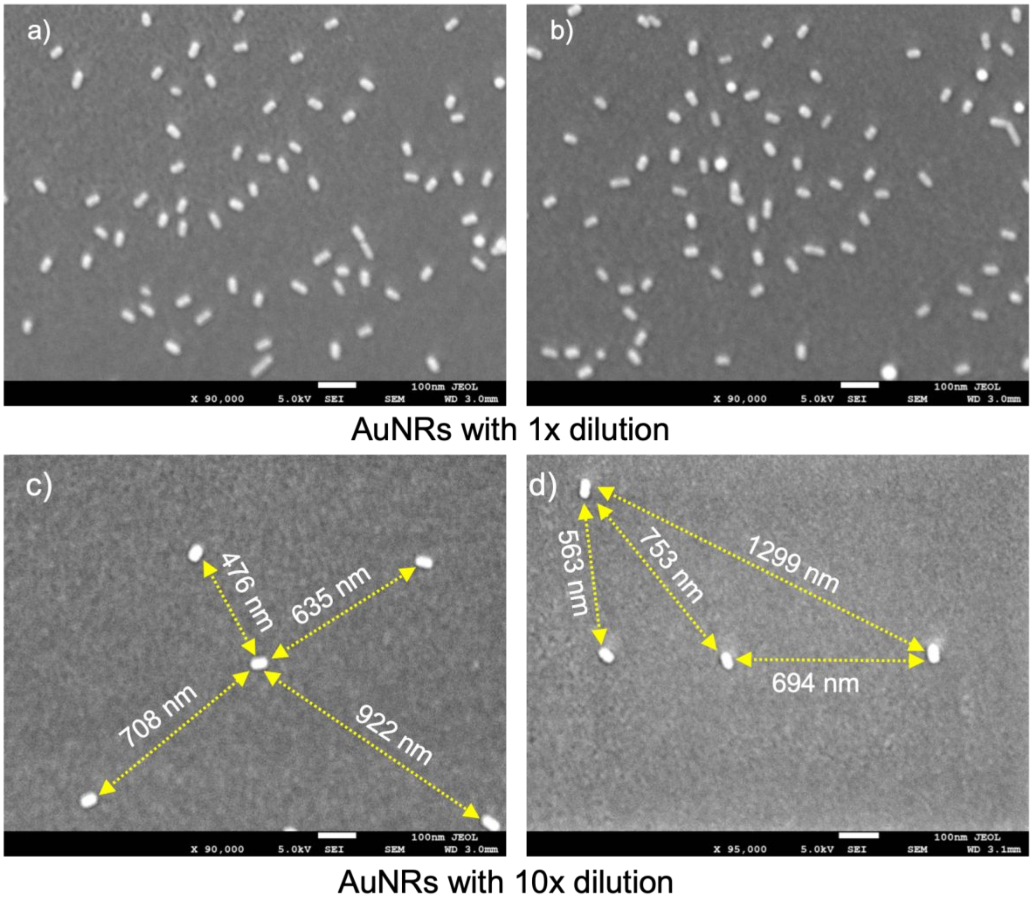


**Figure S2:** SEM images of the substrate preparation approach for 60 nm × 25 nm AuNRs showing morphology and size distribution. (a,b) At 1x dilution, AuNRs are closely spaced which could allow multiple particles to fall within the laser focal spot. (c,d) At 1x dilution, images confirm well isolated AuNRs, which are essential for consistent plasmonic heating.

**Synthesis of $MAPbBr_3$ precursor solution for laser-induced crystallization**

The precursor solution of $MAPbBr_3$ was prepared by dissolving them in a polar solvent DMF. Equimolar amount of precursor salts, MABr (111.97 mg, 1.0 mmol) and $PbBr_2$ (367.01 mg, 1.0 mmol), were first dissolved in 1 mL DMF solution at room temperature. The precursor salts were dissolved by stirring the mixture at 1000 rpm for 2 hours at room temperature to obtain a clear precursor solution. Because of the retrograde solubility characteristic, the precursor salts dissolved in solvents formed supersaturated $MAPbBr_3$ precursor solution at room temperature. For the laser-induced crystallization of $MAPbBr_3$ crystals, precursor solution of $MAPbBr_3$ was mixed with GBL solution in 1:1 (v:v) ratio to obtain an unsaturated solution. Subsequently, 5 μL of the unsaturated solution was taken inside the reaction chamber and sealed with another cover slip to prevent natural

solvent evaporation. After placing the solution, the reaction chamber was placed onto the microscope for crystallization.

**Synthesis of $MAPbBr_3$ microcrystals via natural solvent evaporation**

The synthesis of $MAPbBr_3$ microcrystals were carried out by the spontaneous solvent evaporation method. In this typical synthesis process, first the precursor solutions of $MAPbBr_3$ were prepared by dissolving them in polar solvents DMF and GBL respectively. To crystallize pure $MAPbBr_3$, precursor solution of $MAPbBr_3$ was mixed with GBL solution in 1:1 (v:v) ratio to obtain an unsaturated solution. A microdroplet (ca. 1 µL) from the supernatant of the unsaturated solution was placed on the cover glass. After placing the solution on the cover glass, the microdroplet was observed under microscope. Within minutes the $MAPbBr_3$ microcrystals started growing inside the droplet through natural solvent evaporation. The prepared crystals were then taken under microscope for imaging and spectral analysis.

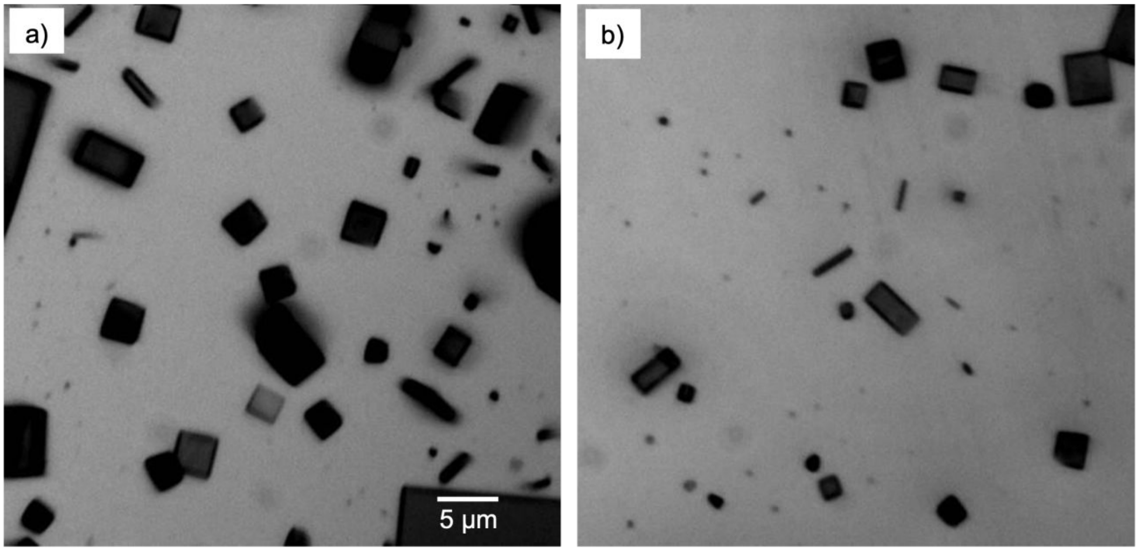


**Figure S3:** Bright-field images of $MAPbBr_3$ crystals formed spontaneously at a precursor concentration of 1.3 M. Uncontrolled nucleation and irregular crystal growth are observed.

## COMSOL Simulations

A commercial finite-element method (FEM) solver, COMSOL Multiphysics v6.2 (www.comsol.com), was employed to calculate the 2D temperature distribution in the medium (DMF) and substrate (Glass). The solver was also used to evaluate the temperature evolution of the nanoparticles under varying laser intensities. The simulations incorporated the computed heat input values to predict temperature profiles under experimental conditions.

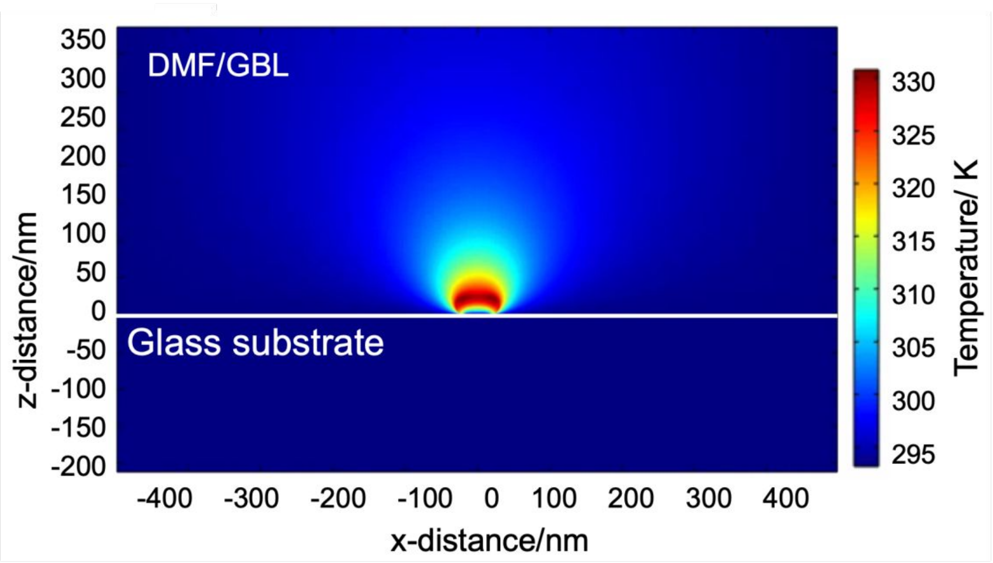


**Figure S4:** Computational 2-D temperature distributions for DMF, GBL/glass

## Laser Intensity Calculation

For a Gaussian beam, the peak laser intensity $I_{laser}$ at the focal point is given by:

$$I_{laser} = \frac{P_{laser}\ (2.3546)^2}{2\pi (FWHM)^2}$$

Using this equation, the laser intensity was calculated to be **4.87×10$^{11}$ W/m²** at the peak.

## Heat Input Calculation

The absorbed heat input (Q) due to laser irradiation is given by:

$$Q = I_{laser} \times C_{abs}$$

where $C_{abs}$ is the absorption cross-section of the nanorod, calculated using Mie theory as **18.43×10$^{-18}$ m²**.

Substituting the values, the heat input was determined to be **8.98×10⁻⁶ W**.

Finite element simulations were performed using COMSOL Multiphysics to model steady-state heat conduction around a gold nanorod (60 nm in length × 25 nm in width) situated at the interface between a borosilicate glass substrate and a solvent medium. The simulations used the Heat Transfer in Solids module in a 2D geometry (x–z plane).

Material thermal conductivities were defined as follows:
• Gold nanorod: 318 $W \cdot m^{-1} \cdot K^{-1}$
• DMF: 0.16 $W \cdot m^{-1} \cdot K^{-1}$
• GBL: 0.18 $W \cdot m^{-1} \cdot K^{-1}$
• Borosilicate glass: 1.1 $W \cdot m^{-1} \cdot K^{-1}$

**Boundary conditions:**
• Temperature continuity and heat flux continuity were enforced at all interfaces: Au–solvent and Au–glass.
• The bottom boundary of the glass substrate and the outer side boundaries of both the solvent and substrate domains were fixed at a temperature (294 K) to simulate thermal equilibrium with the environment.
• The top boundary of the solvent was also held at 294 K.
• Heat loss through the nanoscale interfacial resistance was considered negligible under steady-state conditions.

Laser heating was modeled as a volumetric heat source within the gold nanorod, approximating absorption from continuous-wave (CW) laser irradiation.

We define supersaturation as S = c / c*(T), where c*(T) is the equilibrium (saturation) solubility at temperature T.

For MAPbBr3 in DMF, Saidaminov et al.[1,2] reported a decrease in solubility from 0.80 ± 0.05 g/mL at approximately 25 °C to 0.30 ± 0.05 g/mL at 80 °C. Converting these values using the molar mass of MAPbBr3 (478.98 g/mol) gives:

- c*(298 K) ≈ 1.67 M
- c*(353 K) ≈ 0.63 M

In contrast, GBL is a significantly poorer solvent for $MAPbBr_3$, with solubility less than 0.05 g/mL across this temperature range. As a result, heating leads to a sharper relative decrease in c*(T), producing stronger supersaturation near heated regions.

This retrograde solubility behavior is supported by prior work on $FAPbBr_3$ in a 1:1 DMF: GBL mixture, where solubility decreases by more than threefold between 20 °C and 100 °C, and inverse-temperature crystallization occurs near 55 °C at a concentration of 1 M.

COMSOL Multiphysics simulations were performed to model the steady-state temperature profile around an Au nanorod under CW laser excitation. Simulations were conducted using both DMF and GBL as the solvent medium to evaluate thermal behavior. Although the experimental system used a 1:1 DMF: GBL mixture, separate simulations with pure DMF ($k = 0.16\ W \cdot m^{-1} \cdot K^{-1}$) and pure GBL ($k = 0.18\ W \cdot m^{-1} \cdot K^{-1}$) showed nearly identical temperature distributions, due to the similarity in thermal conductivities. As a result, the temperature at the gold nanorod surface in the mixed solvent can be reliably approximated using either case and the simulation results are considered valid for interpreting supersaturation dynamics in the mixed solvent system.

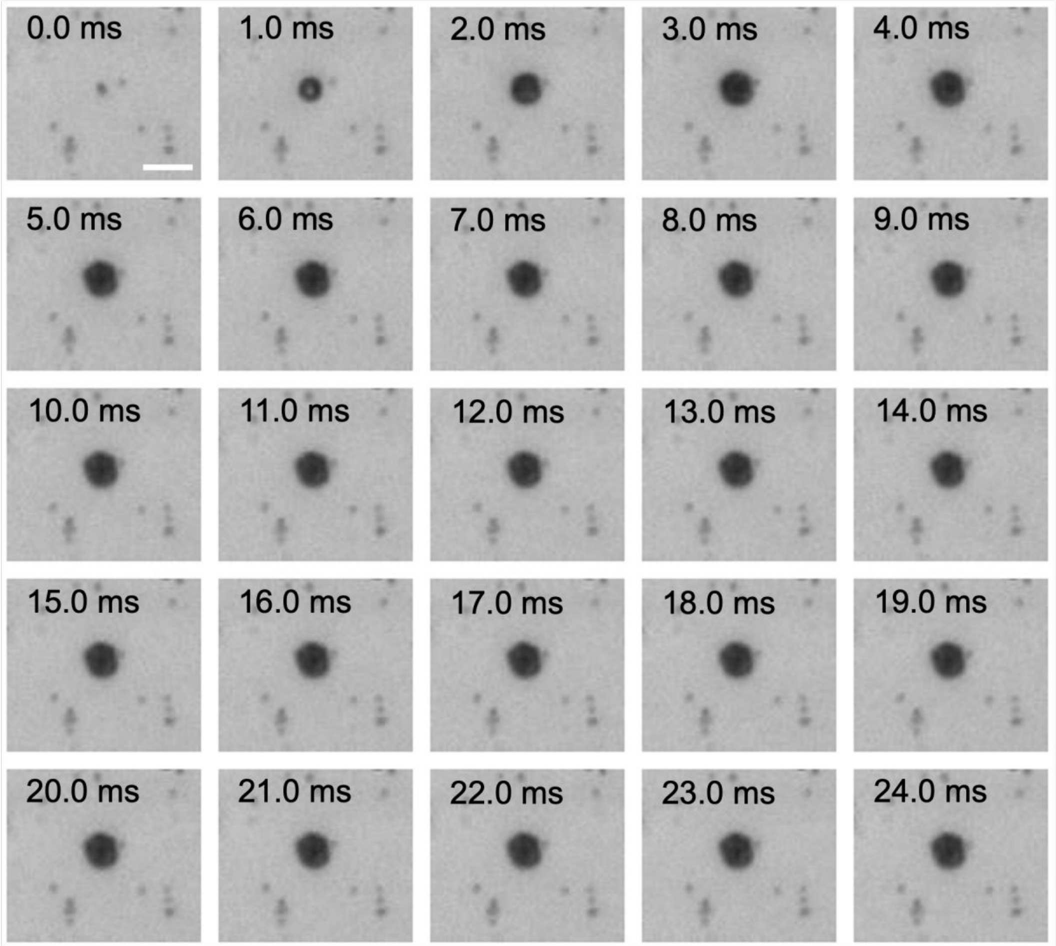


**Figure S5:** A time-lapse of bright-field images showing the progression of $MAPbBr_3$ crystal growth from 0 to 24 ms, at 1-ms intervals. Scale bar 3 μm.

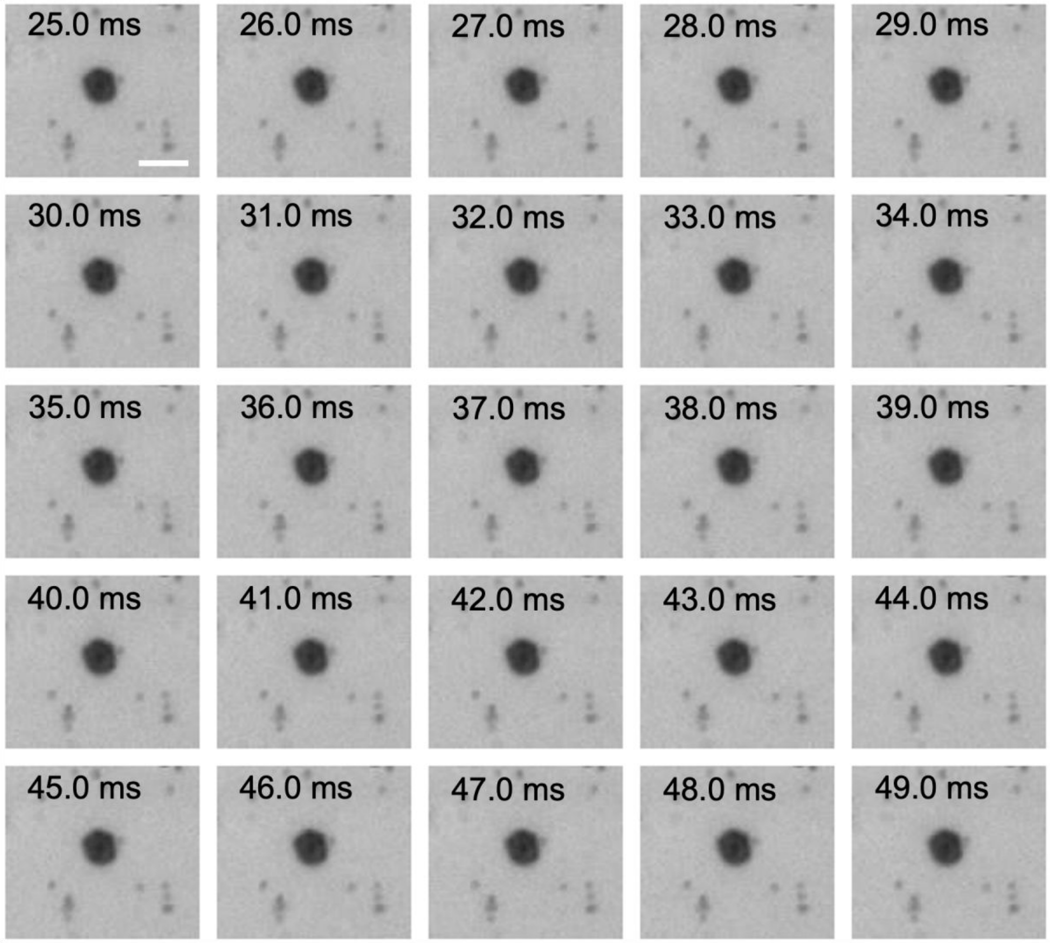


**Figure S6:** A time-lapse of bright-field images showing the progression of $MAPbBr_3$ crystal growth from 25 to 49 ms, at 1-ms intervals. Scale bar 3 μm.

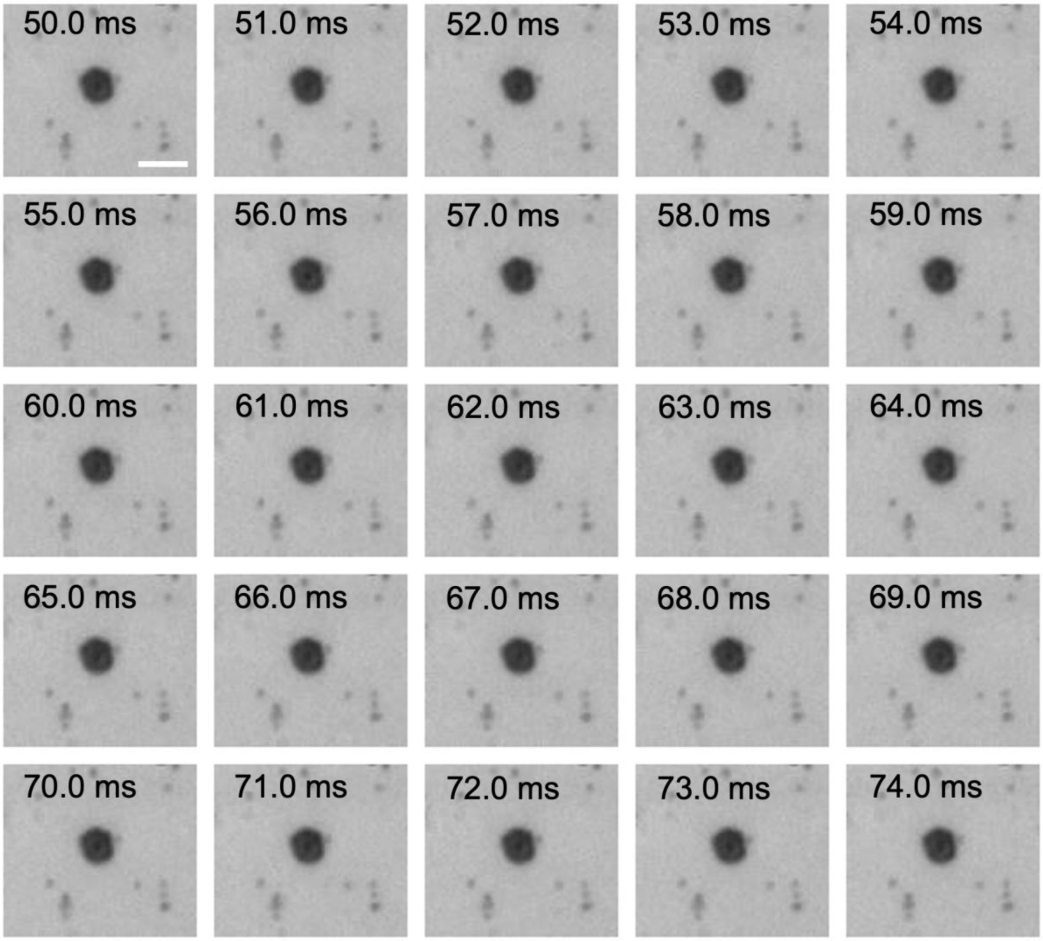


**Figure S7:** A time-lapse of bright-field images showing the progression of $MAPbBr_3$ crystal growth from 50 to 74 ms, at 1-ms intervals. Scale bar 3 μm.

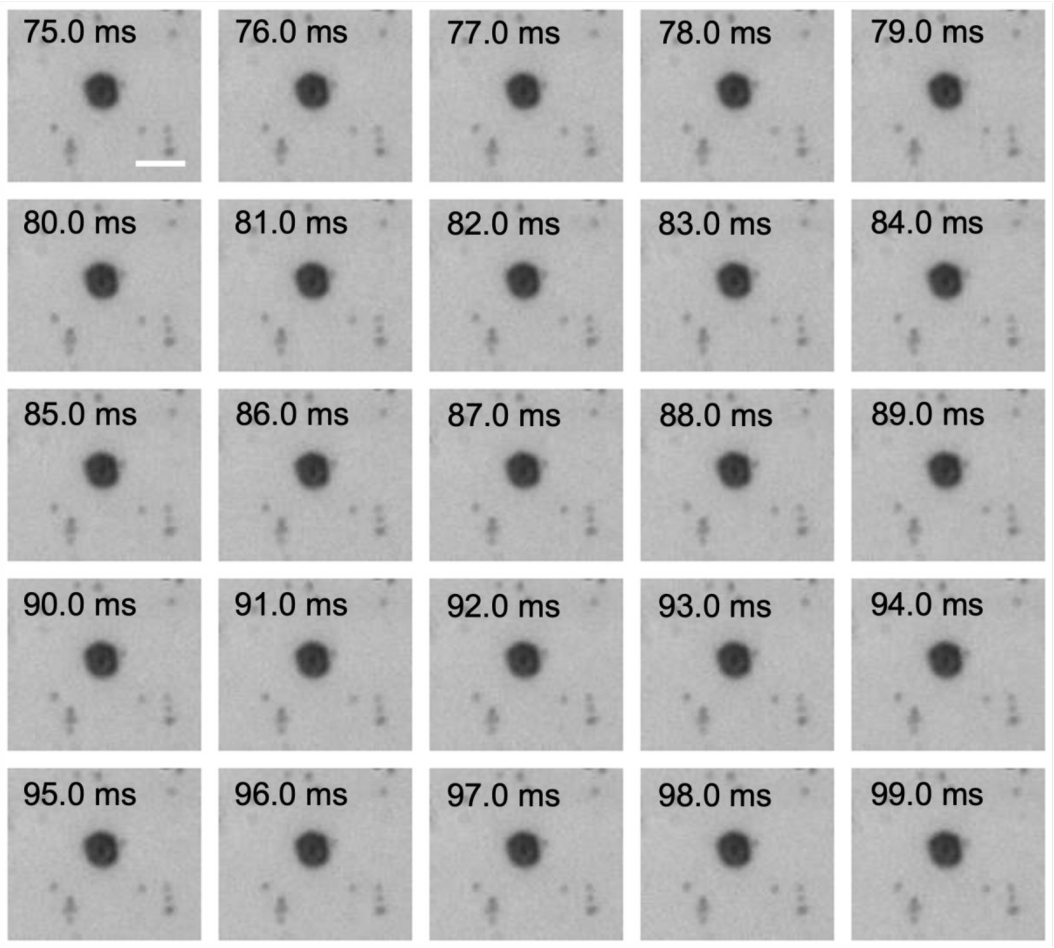


**Figure S8:** A time-lapse of bright-field images showing the progression of $MAPbBr_3$ crystal growth from 75 to 99 ms, at 1-ms intervals. Scale bar 3 μm.

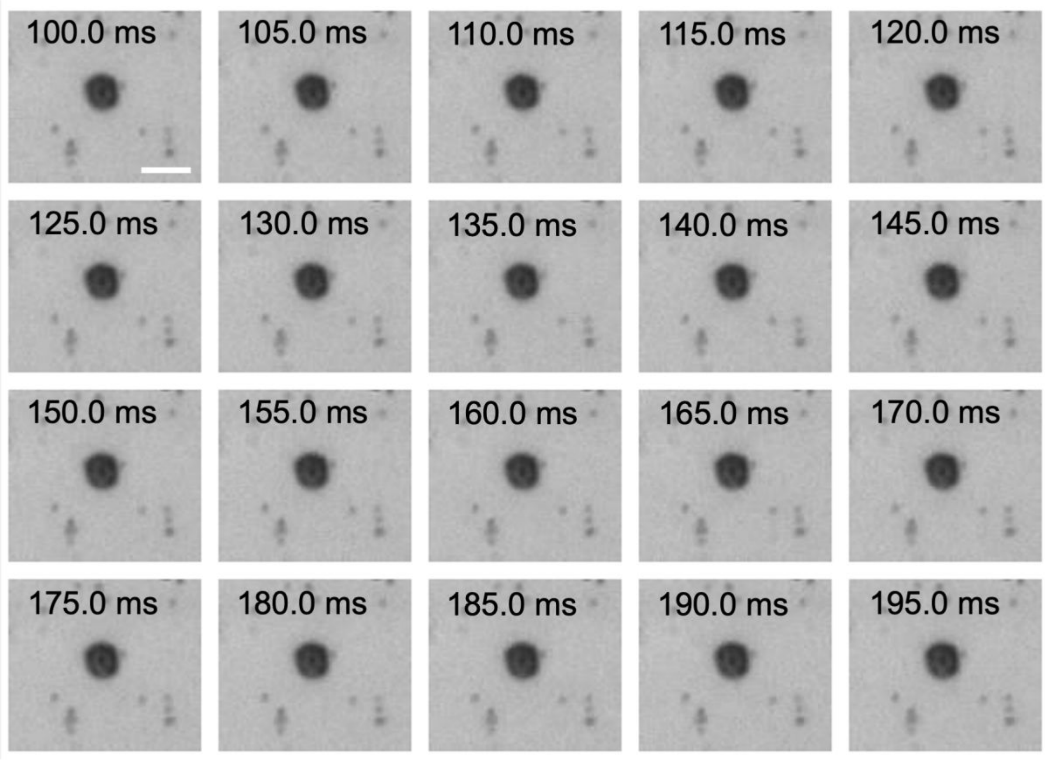


**Figure S9:** A time-lapse of bright-field images showing the progression of $MAPbBr_3$ crystal growth from 100 to 195 ms, at 5-ms intervals. Scale bar 3 μm.

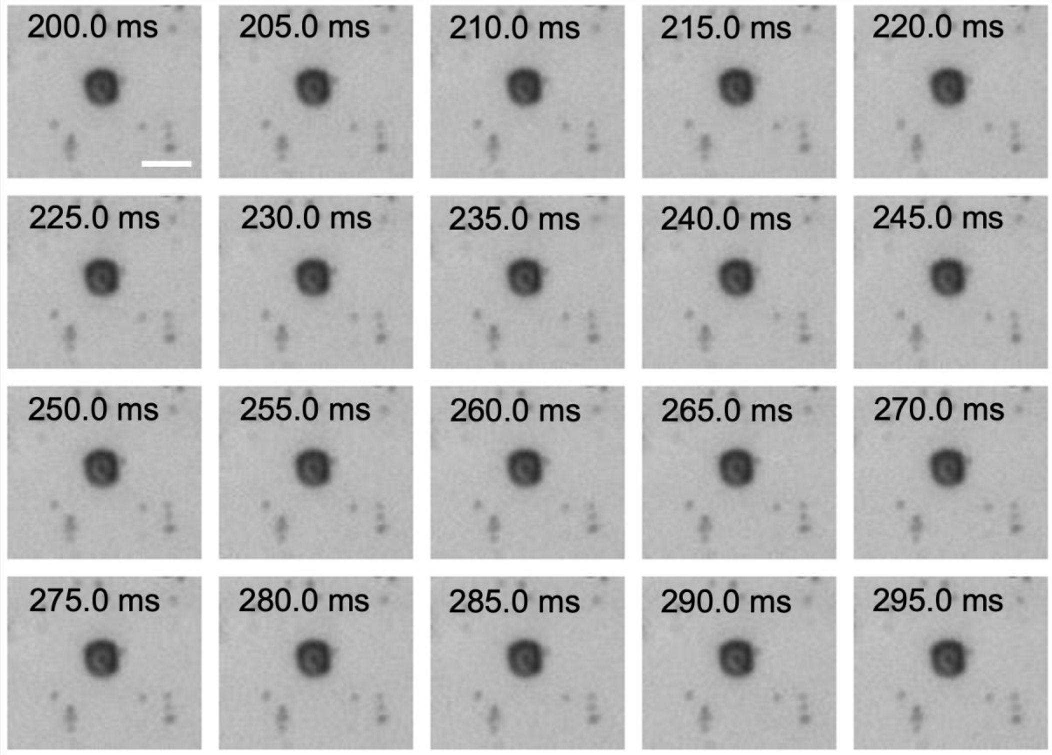


**Figure S10:** A time-lapse of bright-field images showing the progression of $MAPbBr_3$ crystal growth from 200 to 295 ms, at 5-ms intervals. Scale bar 3 μm.

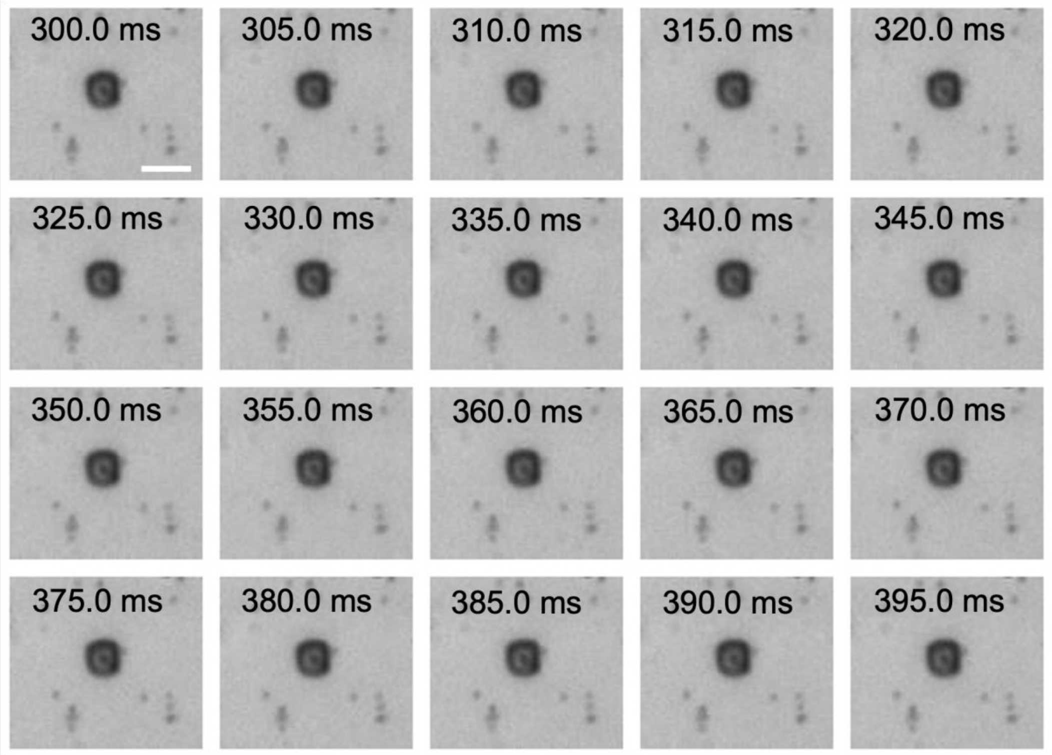


**Figure S11:** A time-lapse of bright-field images showing the progression of $MAPbBr_3$ crystal growth from 300 to 395 ms, at 5-ms intervals. Scale bar 3 μm.

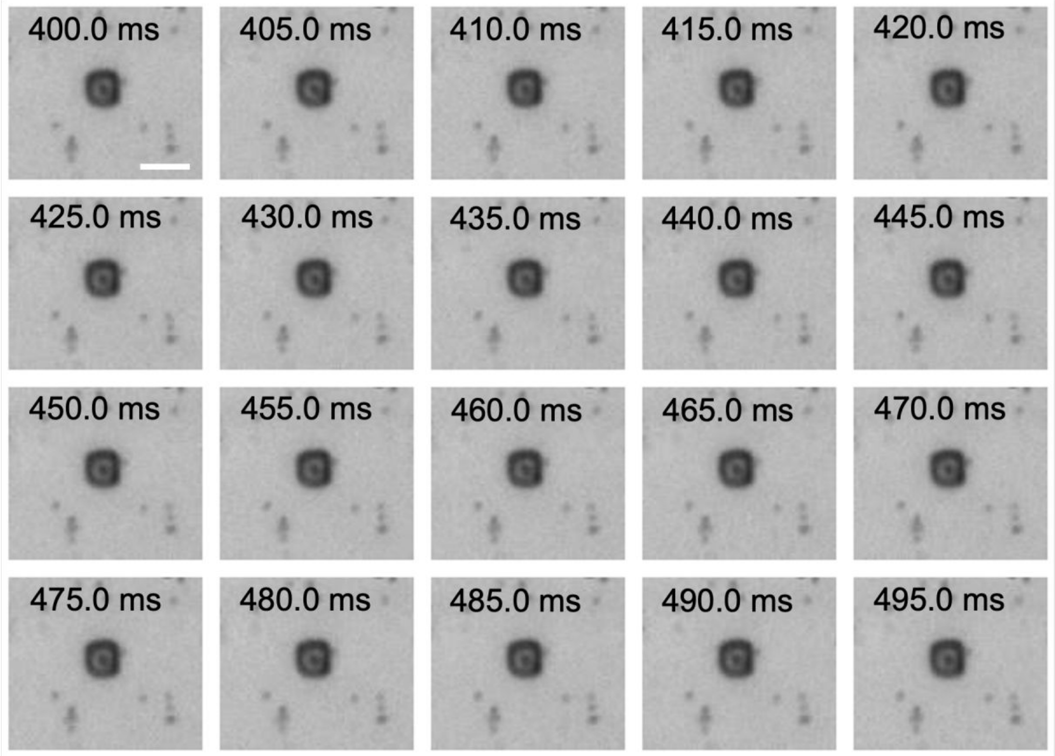


**Figure S12:** A time-lapse of bright-field images showing the progression of $MAPbBr_3$ crystal growth from 400 to 495 ms, at 5-ms intervals. Scale bar 3 μm.

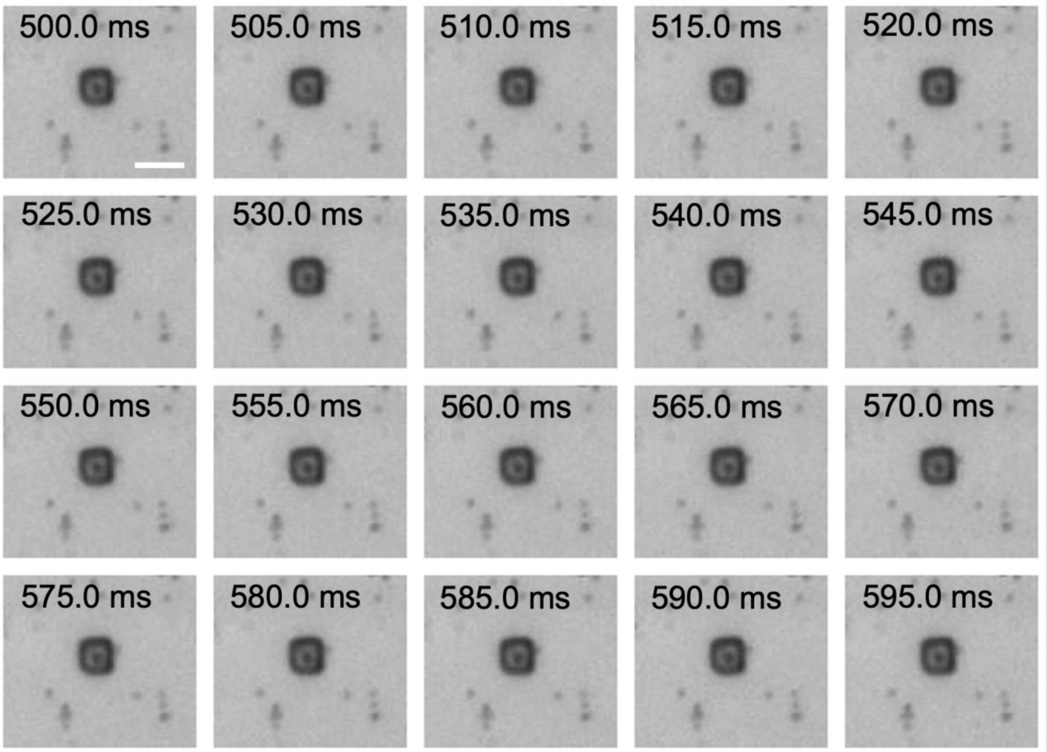


**Figure S13:** A time-lapse of bright-field images showing the progression of $MAPbBr_3$ crystal growth from 500 to 595 ms, at 5-ms intervals. Scale bar 3 μm.

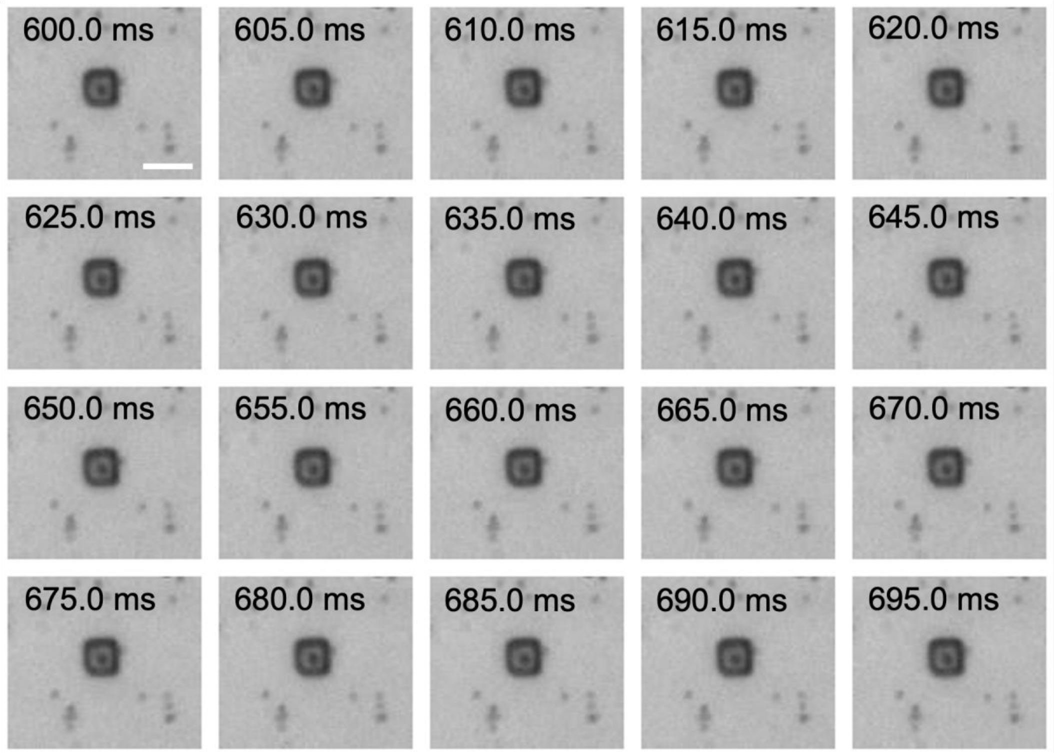


**Figure S14:** A time-lapse of bright-field images showing the progression of $MAPbBr_3$ crystal growth from 600 to 695 ms, at 5-ms intervals. Scale bar 3 μm.

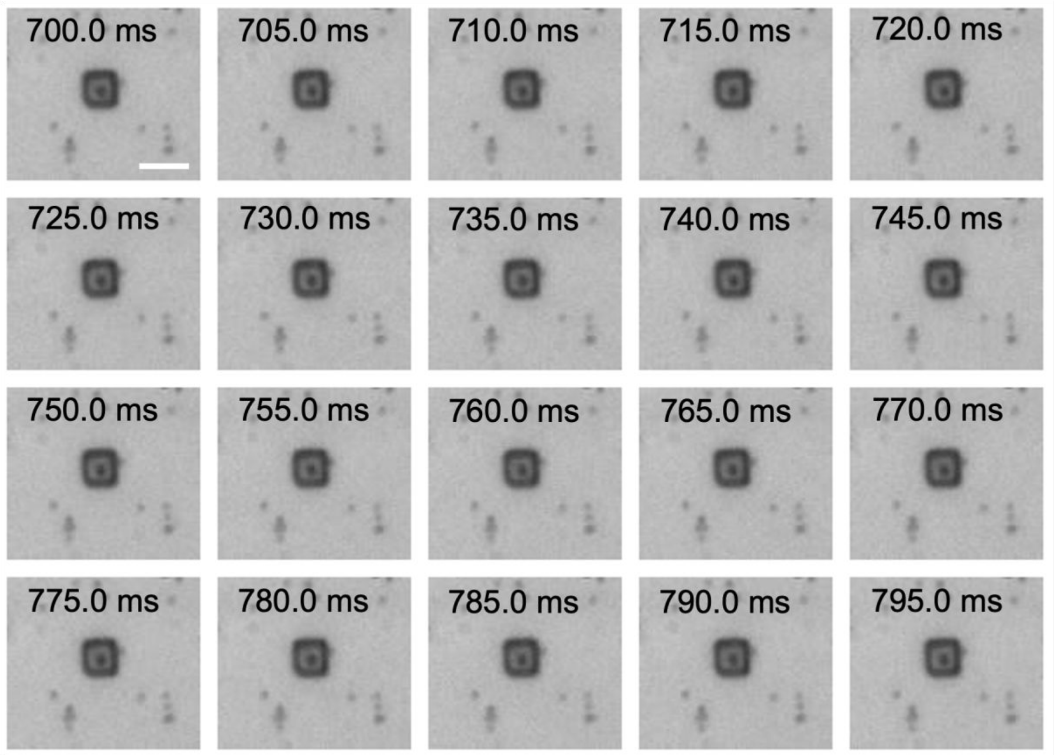


**Figure S15:** A time-lapse of bright-field images showing the progression of $MAPbBr_3$ crystal growth from 700 to 795 ms, at 5-ms intervals. Scale bar 3 μm.

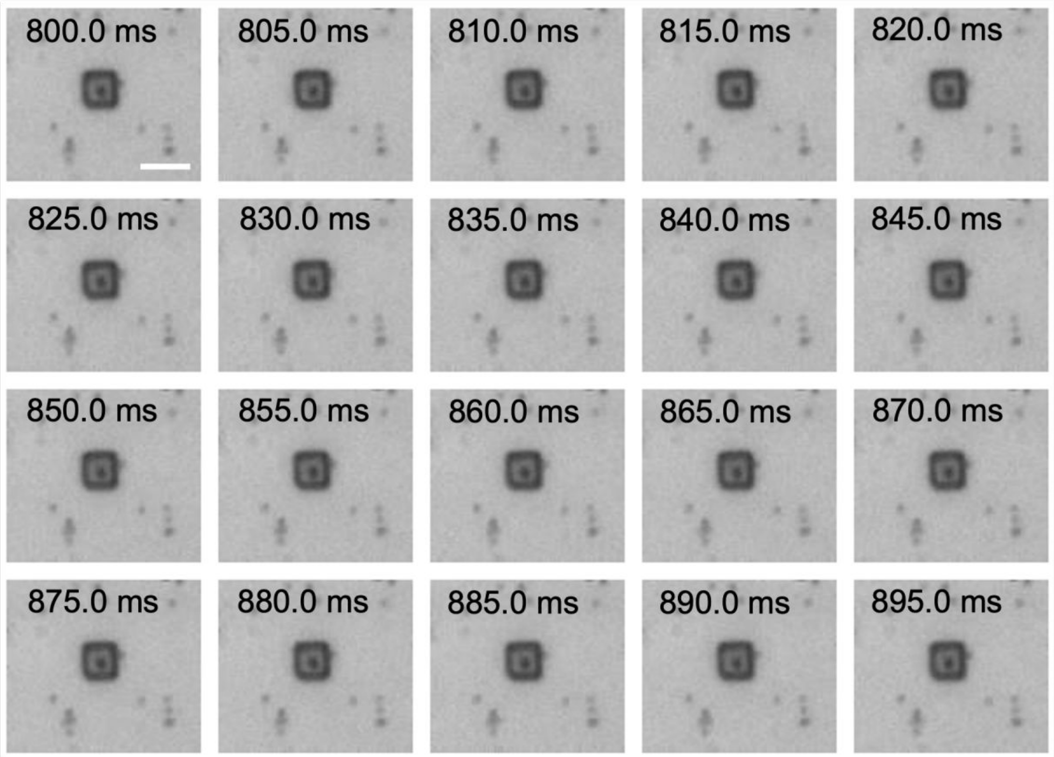


**Figure S16:** A time-lapse of bright-field images showing the progression of $MAPbBr_3$ crystal growth from 800 to 895 ms, at 5-ms intervals. Scale bar 3 μm.

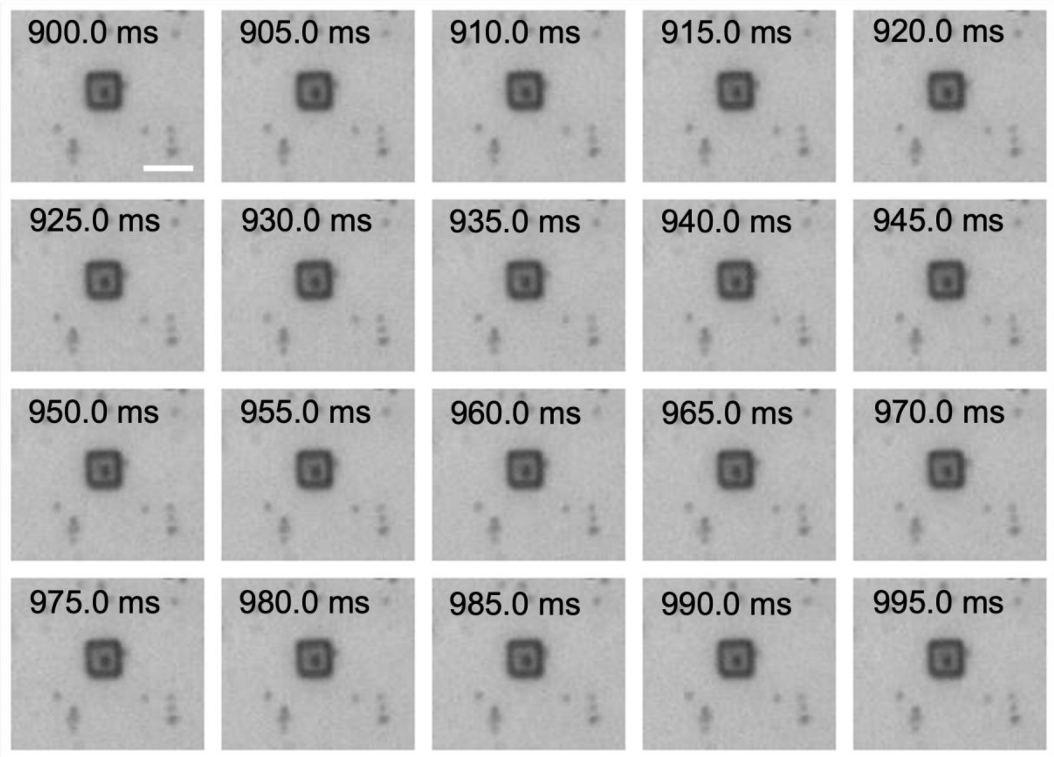


**Figure S17:** A time-lapse of bright-field images showing the progression of $MAPbBr_3$ crystal growth from 900 to 995 ms, at 5-ms intervals. Scale bar 3 μm.

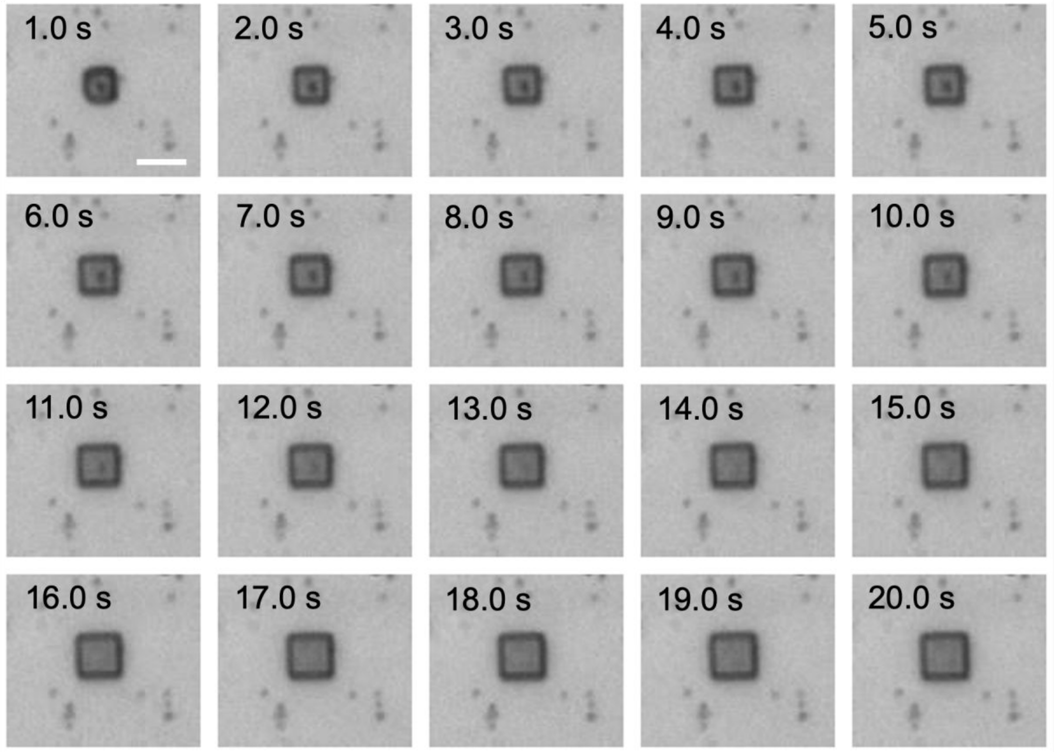


**Figure S18:** A time-lapse of bright-field images showing the progression of $MAPbBr_3$ crystal growth from 1 to 20s, at 1-s intervals. Scale bar 3 μm.

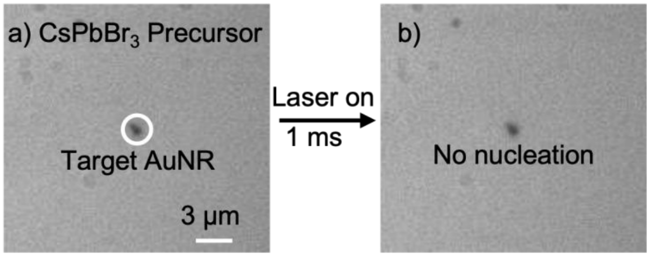


**Figure S19:** Control experiments with $CsPbBr_3$ precursor solution in DMF/GBL under 660nm laser illumination on AuNR. (a) Targeted AuNR under the laser focal spot. (b) No nucleation is observed under identical conditions that produce $MAPbBr_3$ crystallization.

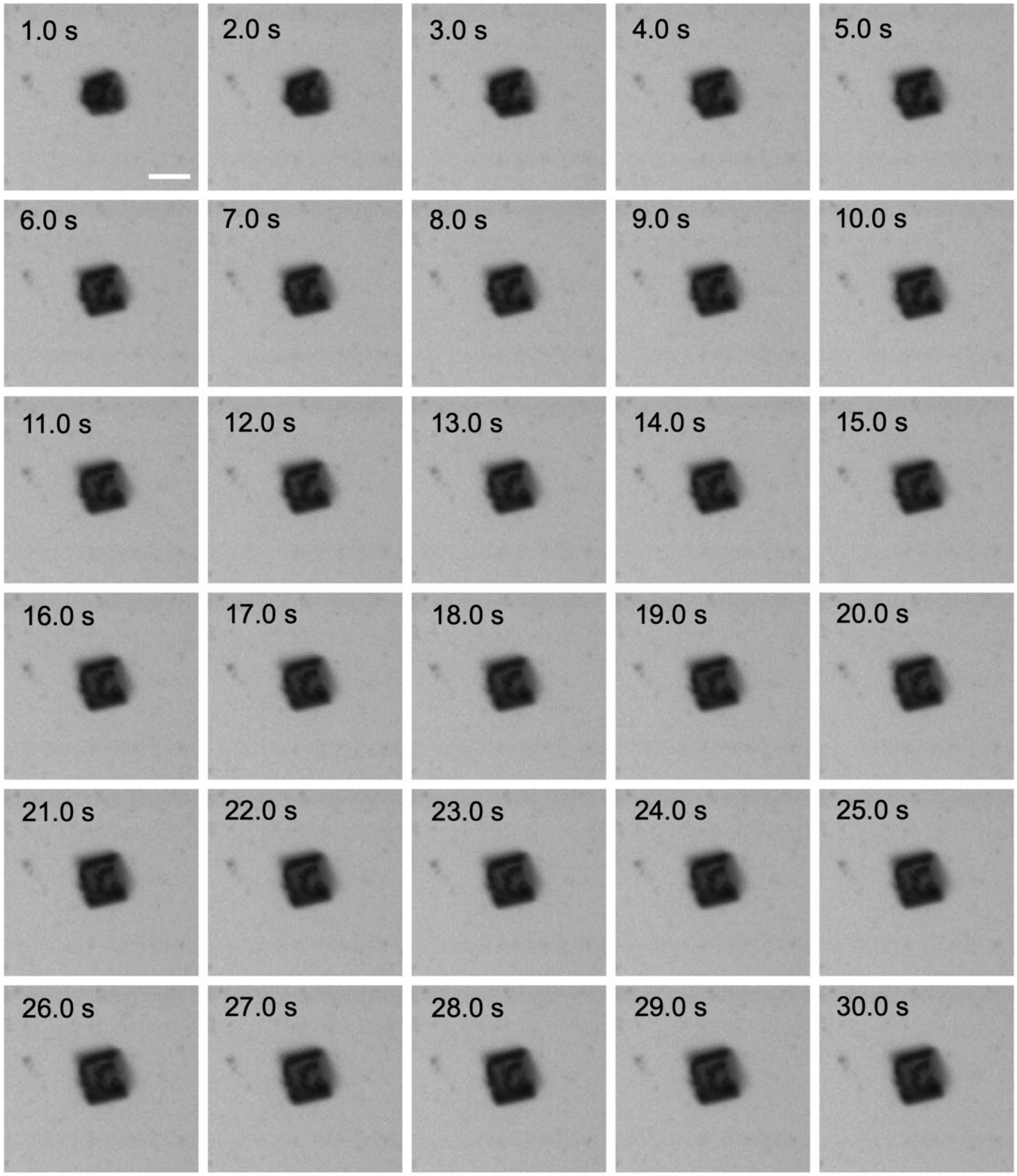


**Figure S20:** A time-lapse of bright-field images showing the progression of $MAPbBr_3$ crystal at 60 mW laser power growth over 30 seconds, at 1-second intervals. Scale bar 3 μm.

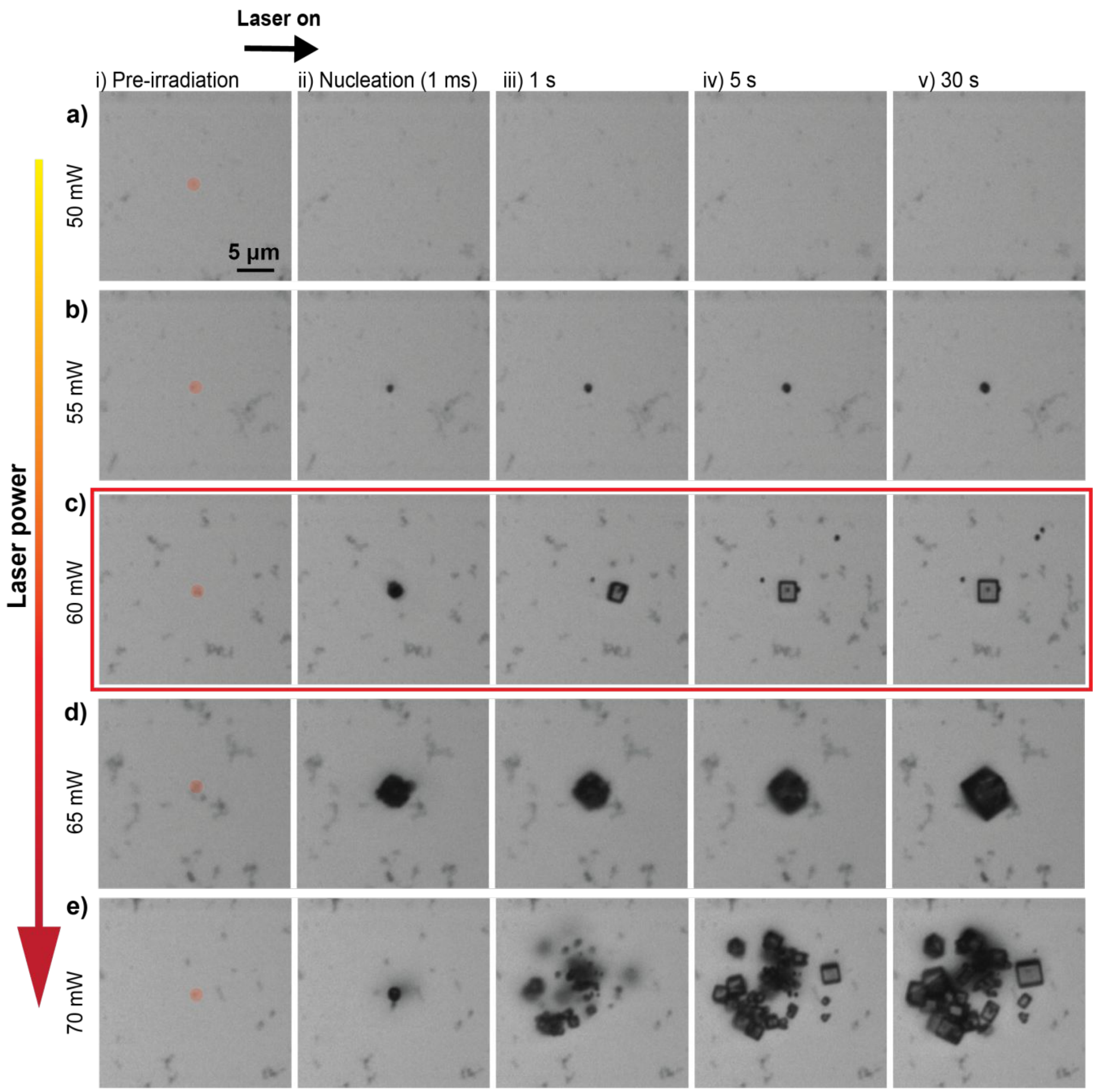


**Figure S21:** Another set of $MAPbBr_3$ crystallization at varying laser power (50-70 mW). (a) No crystallization at 50 mW. (b) Limited nucleation at 55 mW with no significant growth. (c) Optimized nucleation and growth at 60 mW. (d) Faster nucleation and growth at 65 mW, resulting in disordered crystals. (e) Uncontrolled nucleation at 70 mW leading to multiple small crystals, illustrating the critical influence of laser power on crystallization. (a-e) (i) Images of the target AuNR (orange circle) in the precursor solution. (ii) Nucleation of $MAPbBr_3$ crystals at the focal spot after laser irradiation. (iii-v) Growth of $MAPbBr_3$ crystals over time, showing the development of crystal size and morphology at 1s, 5s and 30s.

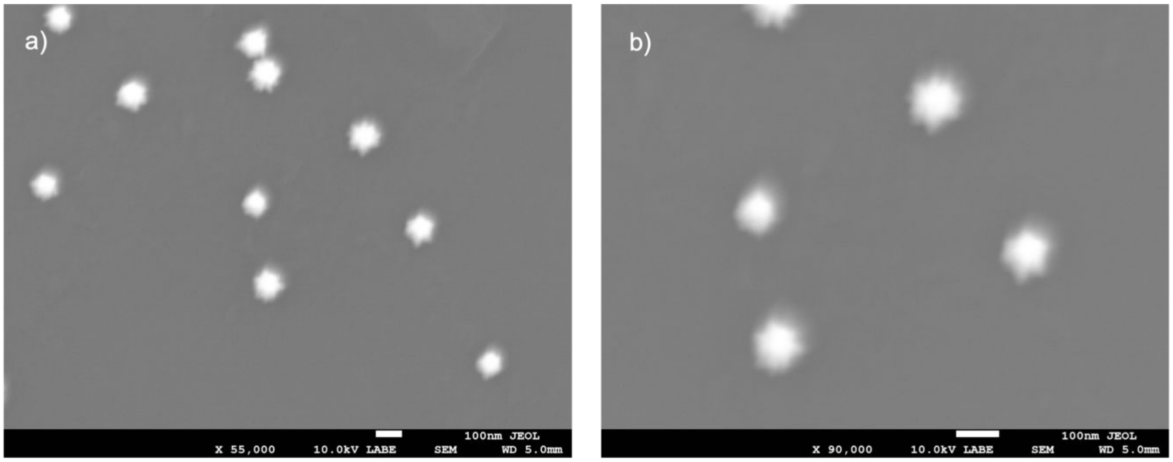


**Figure S22:** SEM images of the substrate prepared with 100nm Au urchins showing morphology and size distribution at different magnification.

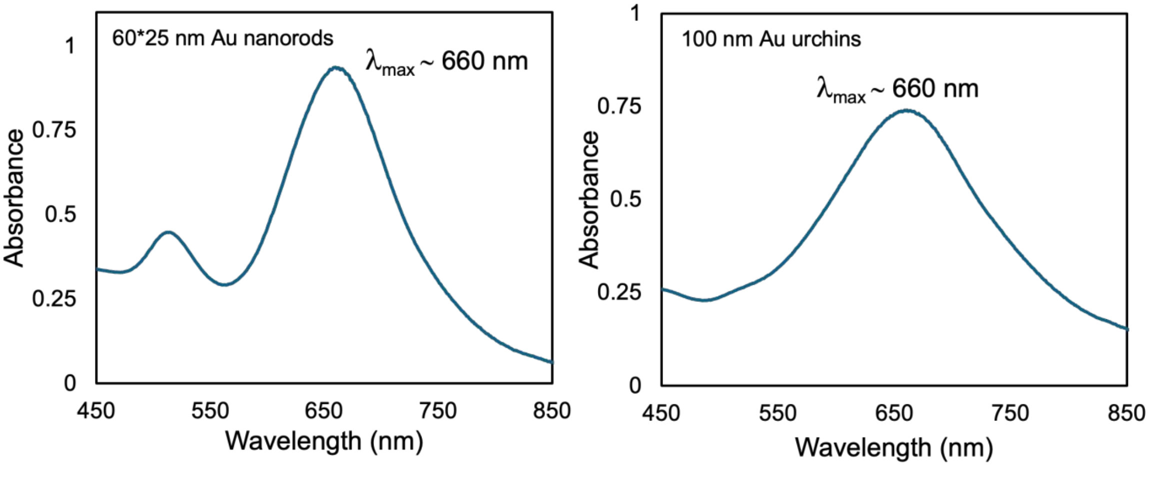


**Figure S23**: Absorption spectra of the 60*25 nm Au nanorods and 100 nm nano urchins. Calculated peak maxima for both the Au nanoparticles are ~ 660 nm.

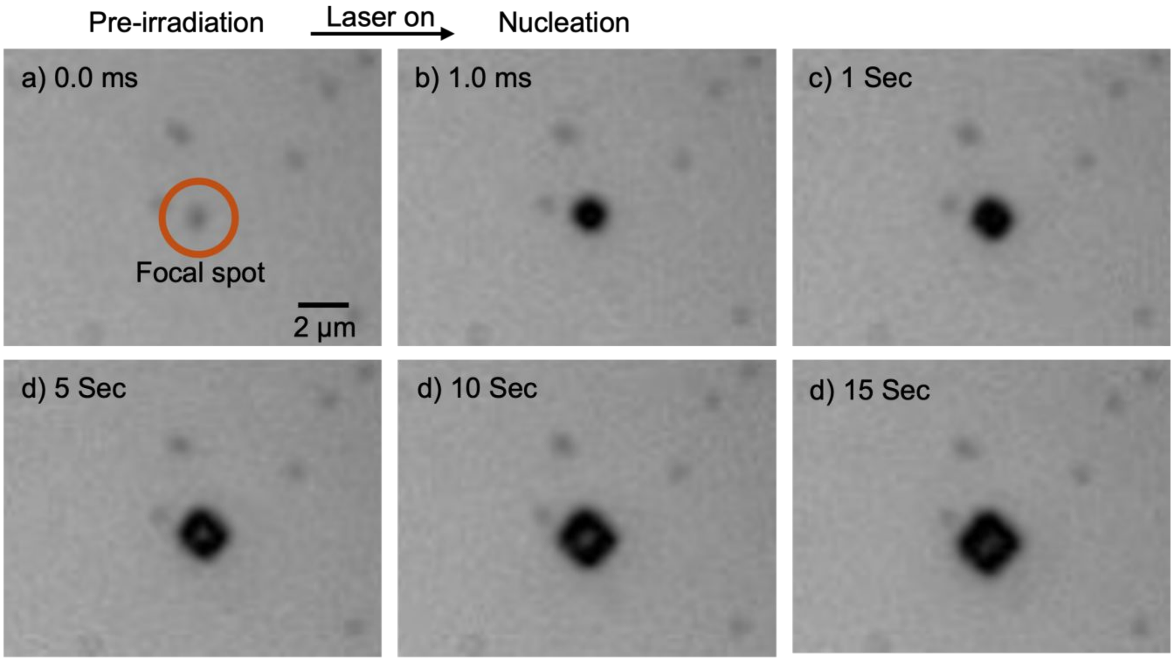


**Figure S24:** A time-lapse of bright-field images showing the crystallization of a $MAPbBr_3$ crystal prepared on 100 nm Au urchins over 15 seconds.

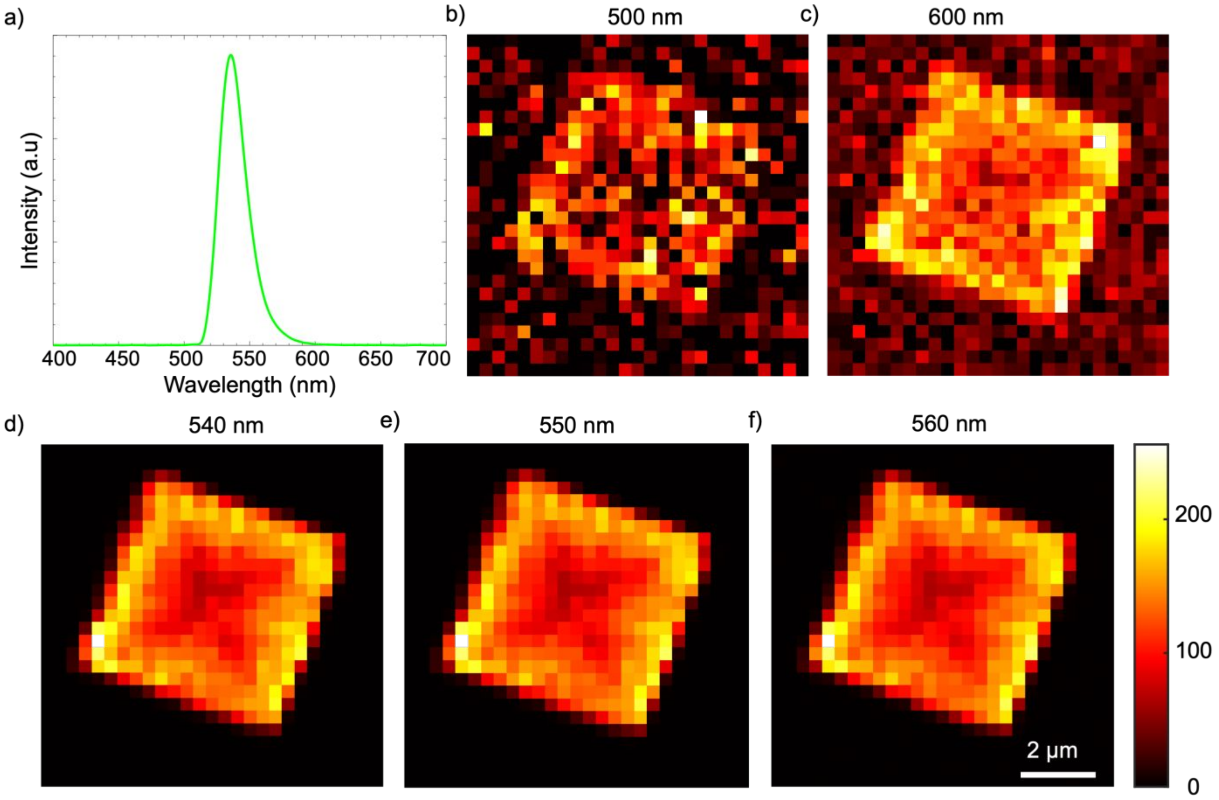


**Figure S25:** Spectral and spatial characterization of a single laser-grown $MAPbBr_3$ crystal using PL mapping. (a) Emission spectrum of the laser-grown crystal, showing a sharp PL peak centered around 535 nm. (b, c) Hyperspectral PL intensity maps at wavelength 500 and 600 nm, revealing poor emission features across the crystal outside the spectral width. (d-f) Spatially resolved intensity maps extracted at three distinct emission wavelengths (540, 550, 560 nm) highlighting edge-enhanced PL intensity observed at peak maxima in laser-induced crystallization of $MAPbBr_3$.

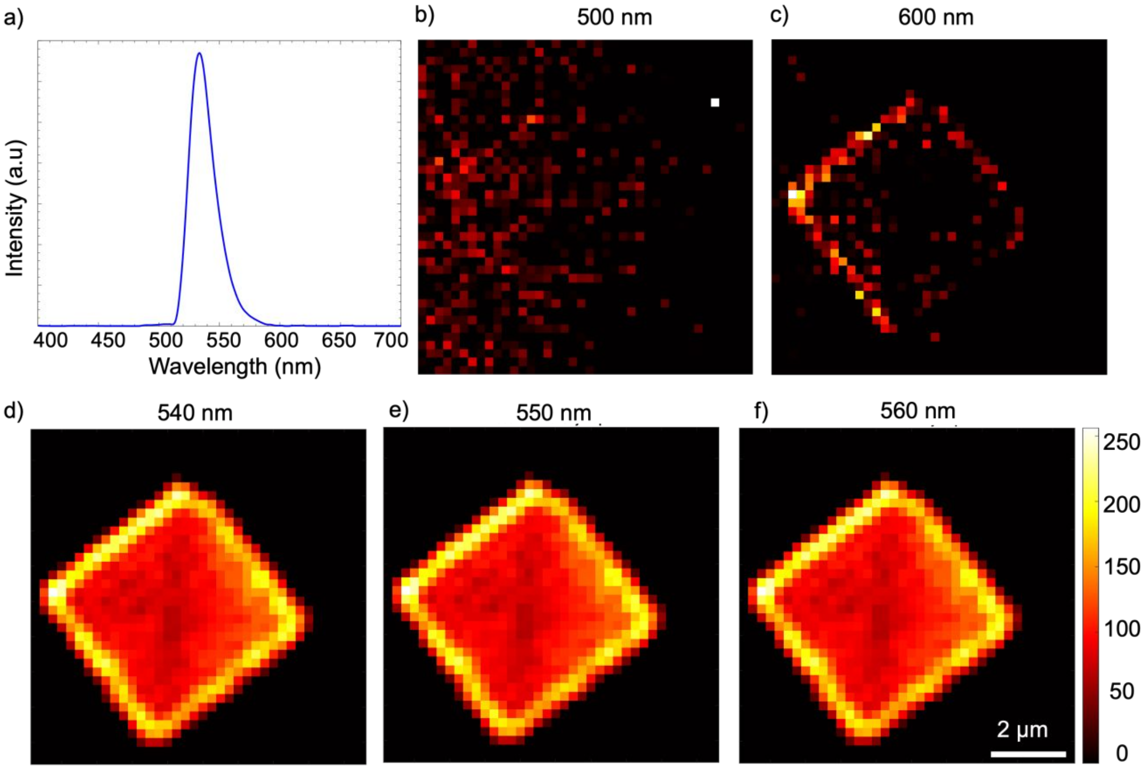


**Figure S26:** Spectral and spatial characterization of a single naturally grown $MAPbBr_3$ crystal using PL mapping. (a) Emission spectrum of the naturally grown crystal, showing a sharp PL peak centered around 533 nm. (b, c) Hyperspectral PL intensity maps at wavelength 500 and 600 nm, revealing poor emission features across the crystal outside the spectral width. (d-f) Spatially resolved intensity maps extracted at three distinct emission wavelengths (540, 550, 560 nm) highlighting edge-enhanced PL intensity observed at peak maxima in natural crystal of $MAPbBr_3$.

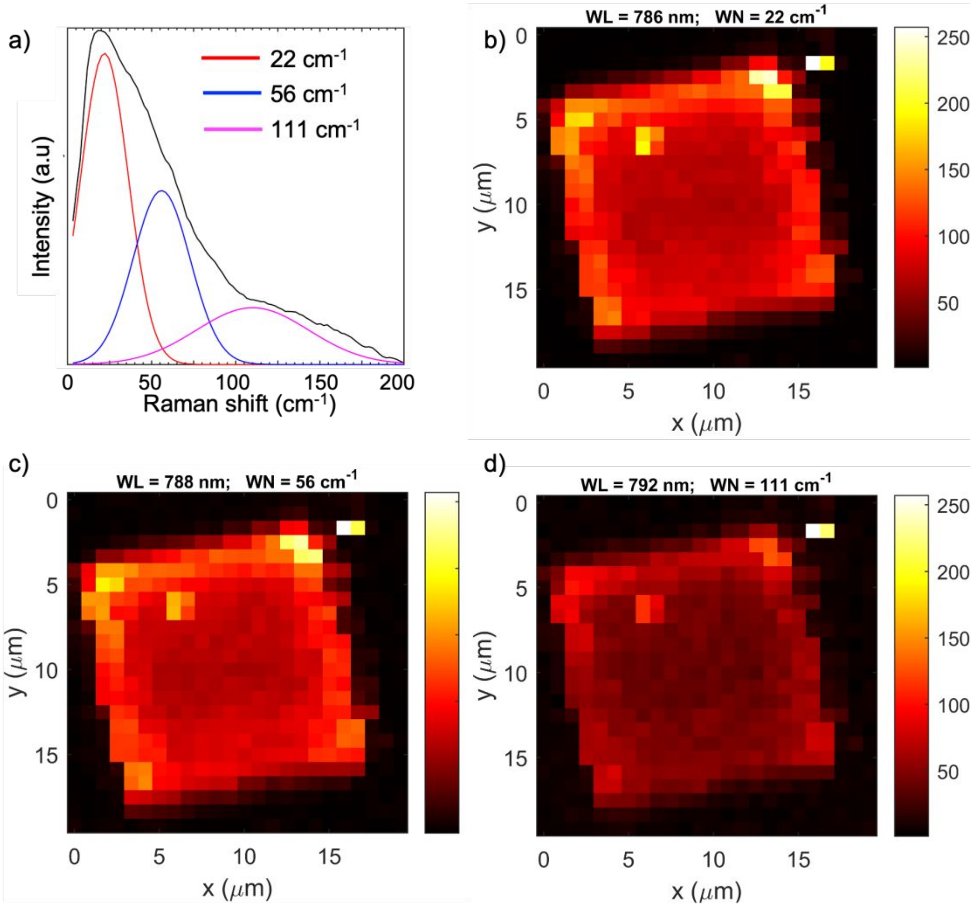


**Figure S27:** Raman spectra and spatial Raman mapping of a naturally grown $MAPbBr_3$ microcrystal. (a) Raman spectrum of the naturally grown $MAPbBr_3$ crystal, with deconvoluted vibrational peaks at 22 $cm^{-1}$ (red), 56 $cm^{-1}$ (blue), and 111 $cm^{-1}$ (magenta). (b-d) Raman intensity maps of the crystal at different vibrational modes: (b) 22 $cm^{-1}$, (c) 56 $cm^{-1}$, and (d) 111 $cm^{-1}$.

References:


1. Saidaminov, M. I.; Abdelhady, A. L.; Murali, B.; Alarousu, E.; Burlakov, V. M.; Peng, W.; Dursun, I.; Wang, L.; He, Y.; Maculan, G.; Goriely, A.; Wu, T.; Mohammed, O. F.; Bakr, O. M. High-Quality Bulk Hybrid Perovskite Single Crystals within Minutes by Inverse Temperature Crystallization. *Nat. Commun.* **2015**, *6*, 7586.
2. Saidaminov, M. I.; Abdelhady, A. L.; Maculan, G.; Bakr, O. M. Retrograde Solubility of Formamidinium and Methylammonium Lead Halide Perovskites Enabling Rapid Single-Crystal Growth. *Chem. Commun*. **2015**, *51*, 17658–17661.
